# Discovery and evolution of an (*R*)-selective transaminase for production of sitagliptin

**DOI:** 10.1039/d6cb00180g

**Published:** 2026-08-20

**Authors:** Matthew Treadell, Gareth R. E. Surman, Grayson J. Ford, Christopher W. Otun, Shiny Joseph Srinivasan, James Galman, Maria Bawn, Akash Pandya, Craig J. Markin, Anthony P. Green, Jack S. Rowbotham

**Affiliations:** a Department of Chemistry, University of Manchester, Manchester Institute of Biotechnology 131 Princess Street Manchester M1 7DN UK jack.rowbotham@manchester.ac.uk; b Johnson Matthey 28 Cambridge science park Cambridge CB4 0FP UK; c Division of Molecular and Cellular Function, University of Manchester, Manchester Institute of Biotechnology 131 Princess Street Manchester M1 7DN UK

## Abstract

We report the discovery and engineering of a new (*R*)-selective transaminase (RTA-223, UniProt W9Z089), identified from *Capronia coronata*. The wild-type enzyme was found to display broad activity on a range of bulky aryl and alkyl ketone substrates, with high enantioselectivity using both d-alanine and isopropyl amine as amine donors. We then investigated activity towards pro-sitagliptin ketone, a well-known challenging pharmaceutical target of industrial significance. Although no forward amination of pro-sitagliptin was initially detected by wild-type RTA-223, low-level activity in the reverse deamination reaction enabled engineering without the need for truncated sitagliptin analogues. Adopting this reverse screening strategy, two active-site mutations unlocked forward amination activity, and subsequent rounds of directed evolution delivered a quadruple mutant (H53L/V60G/F113A/V148A) capable of converting pro-sitagliptin to (*R*)-sitagliptin with high levels of stereoselectivity (e.r. 93 : 7). We further performed a kinetic analysis of the candidates from across the evolution process, using a previously reported coupled assay system linked to d-alanine oxidation. This work validates reverse screening as an effective approach for evolving transaminases toward sterically hindered substrates and highlights RTA-223 as a promising candidate for further biocatalyst development.

## Abstract 2

A new (*R*)-selective transaminase (RTA-223, UniProt W9Z089) is identified from *Capronia coronata* with activity towards a range of bulky ketone substrates. We demonstrate the directed evolution of the enzyme for production of the challenging pharmaceutical target (*R*)-sitagliptin.

## Graphical abstract

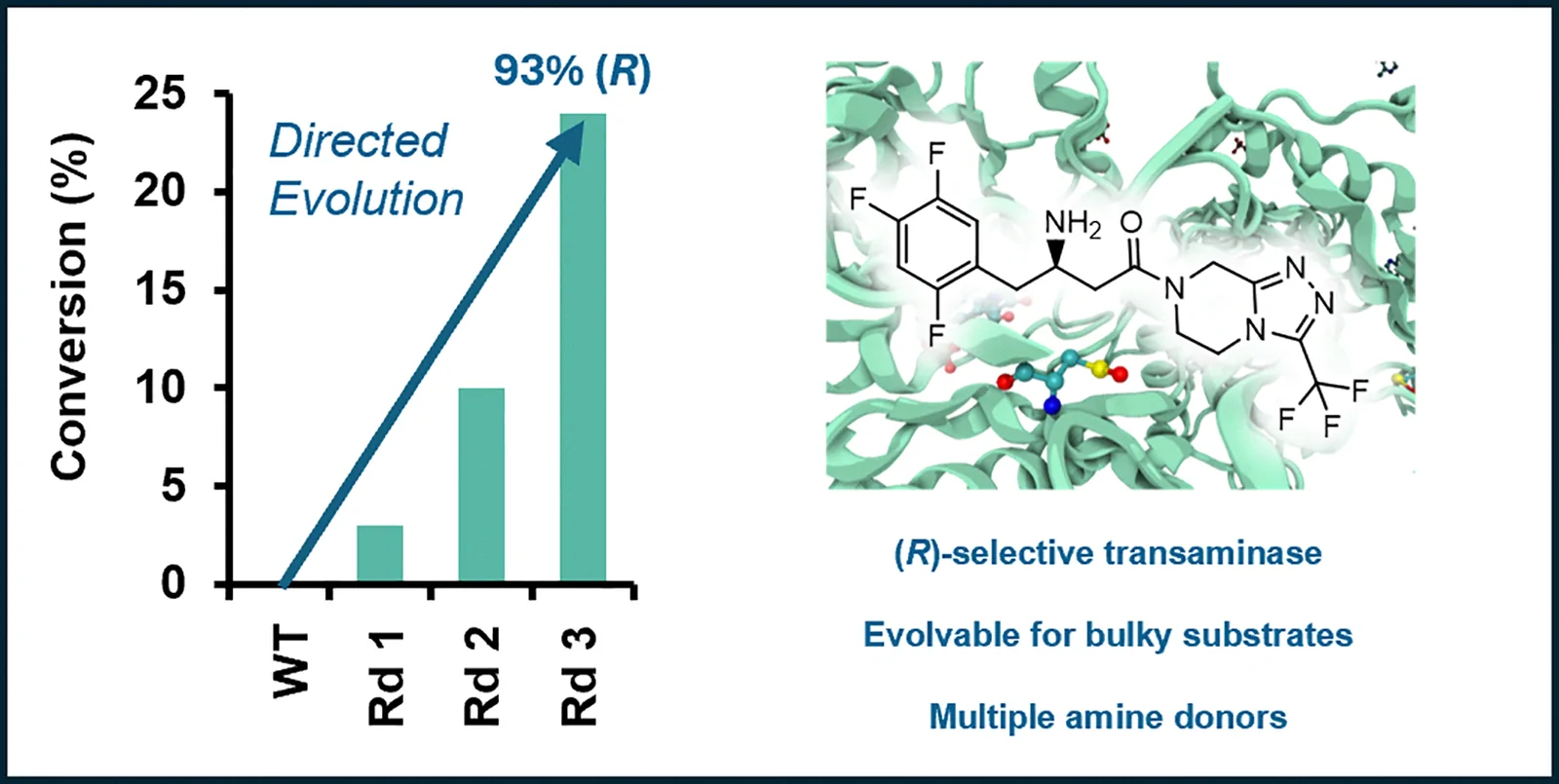

## Introduction

Chiral amines are a key functional group in pharmaceuticals, appearing in 42% of the top 200 drugs in 2022,^1^ highlighting the need for scalable stereoselective routes to their synthesis. Related to this, the biocatalytic toolbox for amine formation has expanded significantly in the last two decades,^2,3^ with enzymatic methods frequently representing a desirable sustainable alternative to more traditional synthetic methods. Here, transaminases (TAs) are known for being robust, evolvable, and possessing a broad substrate scope.^4–6^

**Scheme 1:**
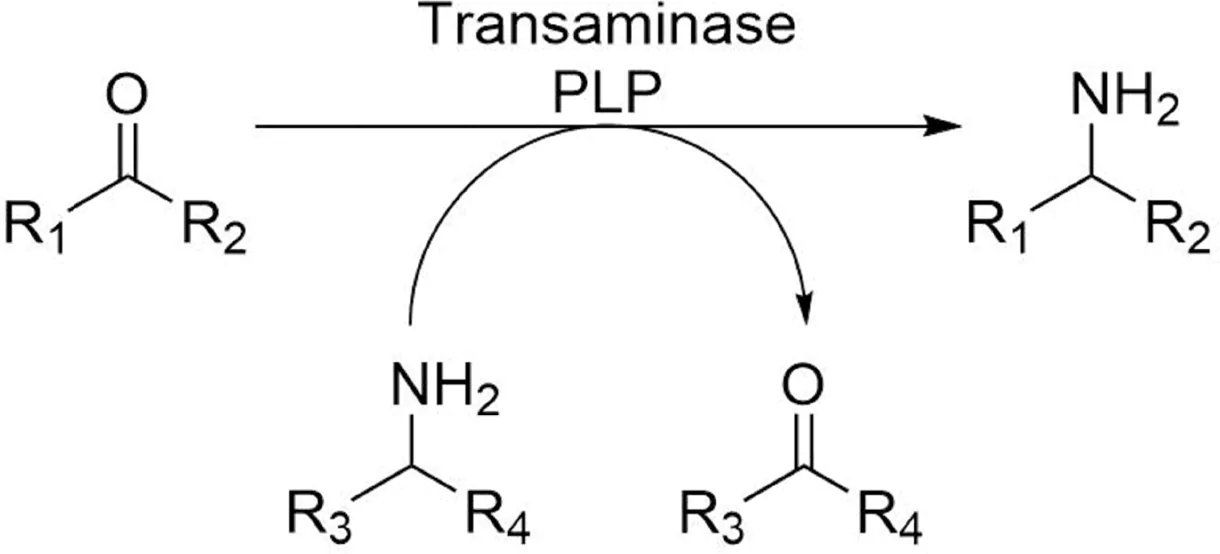
Generalised transaminase reaction.

TAs are pyridoxal 5′-phosphate (PLP)-dependent enzymes that carry out stereoselective transfer of an amine group from a donor molecule to a ketone acceptor (Scheme 1). Their industrial relevance is now well established, with multiple evolution campaigns being conducted to produce enzymes for pharmaceutical manufacturing.^4,7–10^ Sterically hindered substrates remain a key challenge for TAs, especially when wild-type (WT) activity is absent, making directed evolution difficult.^11–13^ There are ongoing efforts to widen the pool of TAs, especially those with broad substrate scope, activity on bulky substrates, and with (*R*)-stereoselectivity (which is notably more scarce in natural sequences).^14–17^ These wider panels of enzymes increase the likelihood for initial positive hits on new amine targets, from which improved activity and selectivity can be evolved.^8^ Additional traits such as thermostability and the ability to use selected amine donors (especially isopropyl amine, IPA) are desirable for industrial applications.^18,19^

With a continually expanding range of bioinformatic and computational tools for identifying and generating new sequences, rapid colorimetric/fluorometric assays are well-placed to overcome bottlenecks and costs associated with high-throughput screening of large enzyme libraries.^20–22^

As such, in this study we investigate a transaminase from *Capronia coronata* (RTA-223), evaluate its substrate scope, and introduce new activity towards (*R*)-sitagliptin, a valuable pharmaceutical amine target and benchmark for the directed evolution of transaminases towards bulky substrates.

## Results and discussion

### Identification of a putative TA and investigation of substrate scope

Rodriguez Giordano and coworkers recently described the characterisation of a transaminase from *Capronia semiimmersa* (CsTA) which displayed both (*R*)-selectivity and the ability to use isopropyl amine as an amine donor.^23^ Given the relative scarcity of natural (*R*)-transaminases and the desirability of IPA as a cheap amine donor, we carried out a BLAST search for similar sequences from the UniProt database.^24^ Three sequences from *Capronia* species were identified; a previously reported transaminase from *C. epimyces* (*Ce*TA, UniProt W9Y937), and two identified from *C. coronata* (W9Z089 and W9XM12). *Ce*TA was previously investigated for d-alanine production from (*R*)-α-methylbenzylamine and was recently shown to be effective for production of a wide range of *trans*-β-amino alcohols.^25,26^ Of the two reported *C. coronata* transaminases, W9Z089 (herein referred to as RTA-223) was chosen as a target due to its relation to *Ce*TA in the phylogenetic tree inferred from BLAST based sequence analysis (Fig. S1). RTA-223 was predicted to be a promising candidate for (*R*)-selective TA activity, as it contains the well documented H****Y*V*H(A/S) and F(Y)VN(S/E) signature regions (Fig. S2).^25^ Whilst RTA-223 displays 83% and 64% sequence identity to *Ce*TA and *Cs*TA respectively, it only displays a 42% sequence similarity to ATA-117, the TA evolved by Merck and Codexis for the formation of (*R*)-sitagliptin.^23^

**Fig. 1:**
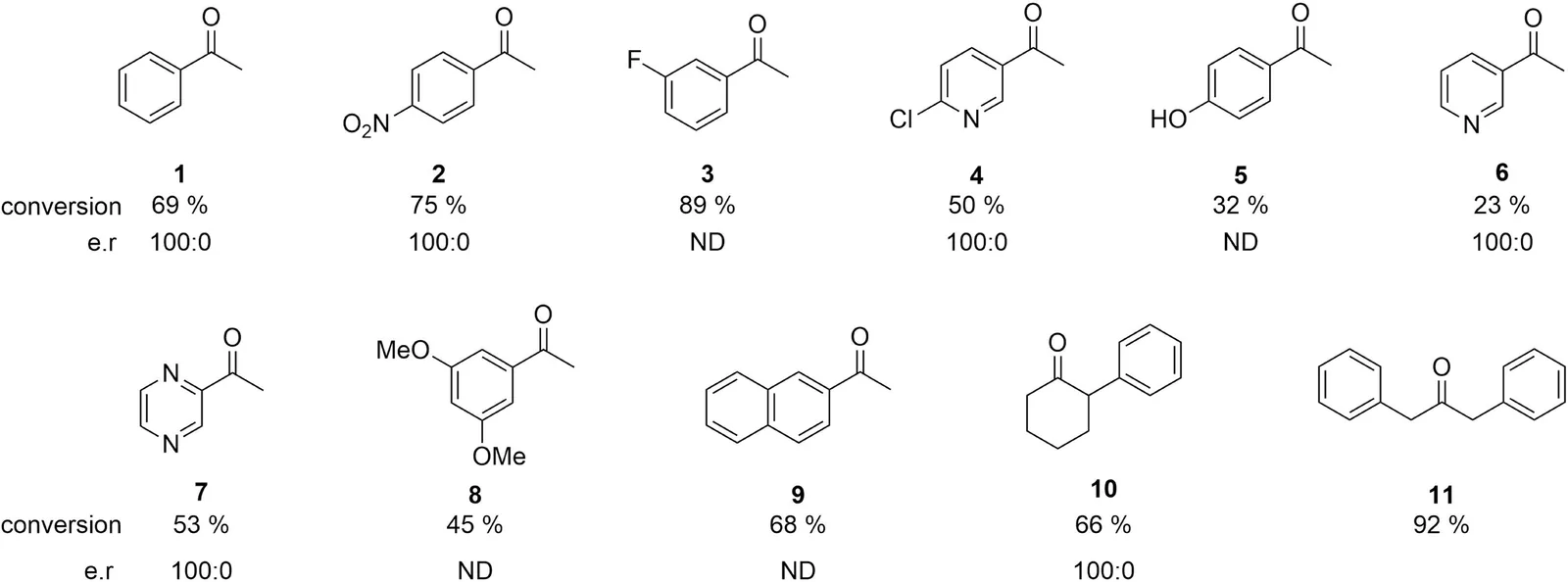
Substrate scope of RTA-223. Conditions: 5 mg mL^−1^ purified RTA-223, 5 mM ketone, 500 mM amine donor (1 – IPA, 2-11 – d-alanine), 100 µM PLP in 5% DMSO, 100 mM triethanolamine at 30 °C for 24 hours.

The starting RTA-223 construct was expressed recombinantly in *E.coli* and purified. Purified RTA-223 was then tested for the reductive amination of acetophenone (1) and gave 69% conversion, by GC-MS analysis, to (*R*)-α-methylbenzylamine with IPA as the amine donor, demonstrating that IPA activity would not need to be introduced with evolution. A number of other acetophenone derivatives (2-9) were also evaluated with d-alanine as the donor, and all were found to have substantial activity and, in the cases tested, complete selectivity for the (*R*)-product (Fig. 1). These results were encouraging, indicating tolerance to common functional groups (alcohols, halides, nitro) as well as pyridine and pyrazine derivatives. Bulky ether and napthyl substrates (8 and 9) were also accepted. We then verified RTA-223 for activity on non-benzylic ketones (10 and 11), with similar success, even in the case of the symmetrically sterically hindered 1,3-diphenylacetone. Following the initial substrate scoping, we decided to investigate the evolvability of RTA-223 towards a target drug compound.

### Initial screening for activity towards (*R*)-sitagliptin and analogues

**Fig. 2:**
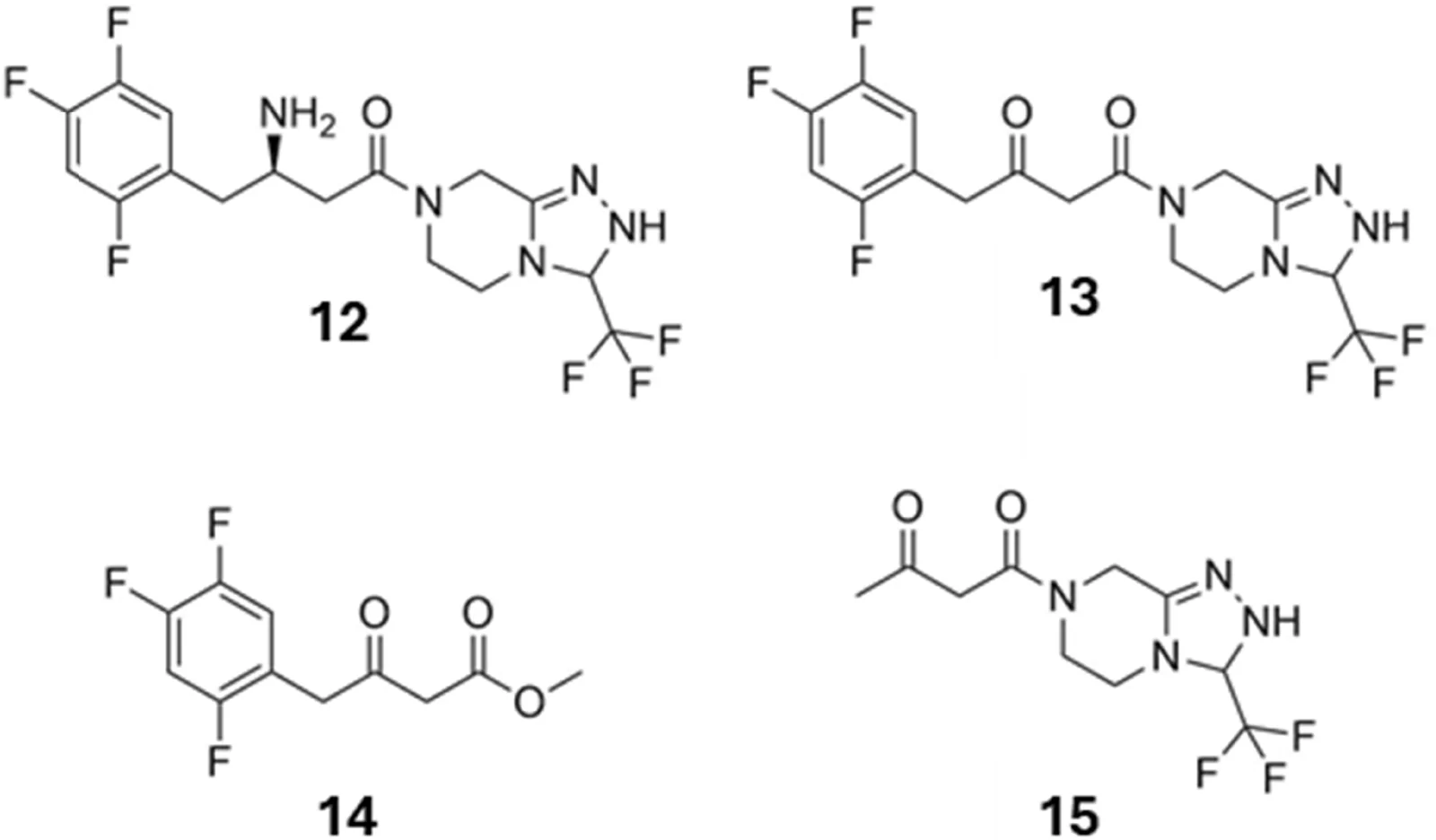
(*R*)-Sitagliptin ((*R*)-12)) and its ketone precursor pro-sitagliptin (13). Compounds 14 and 15 are truncated forms of 13, with 15 used in a previous study to evolve a TA with activity towards the formation of (*R*)-12.

To explore the potential of RTA-223 for stereoselective amination of sterically hindered ketone drug targets, the antidiabetic (*R*)-sitagliptin (Fig. 2, (R)-12) was selected as a synthetic target.^27–30^ (*R*)-Sitagliptin has been a popular drug target to benchmark TA evolution against and multiple candidates have been reported in the academic and patent literature since the original ATA-117 campaign.^7,31–33^ Some of these TA homologues have been included in the supplementary bioinformatic analysis (Fig. S1 and S2) to allow comparison with RTA-223. In the evolution of ATA-117, the initial round of mutagenesis focused on a truncated pro-sitagliptin substrate 15, before expanding to the full molecule 13 in a substrate walking approach.^7^

**Fig. 3:**
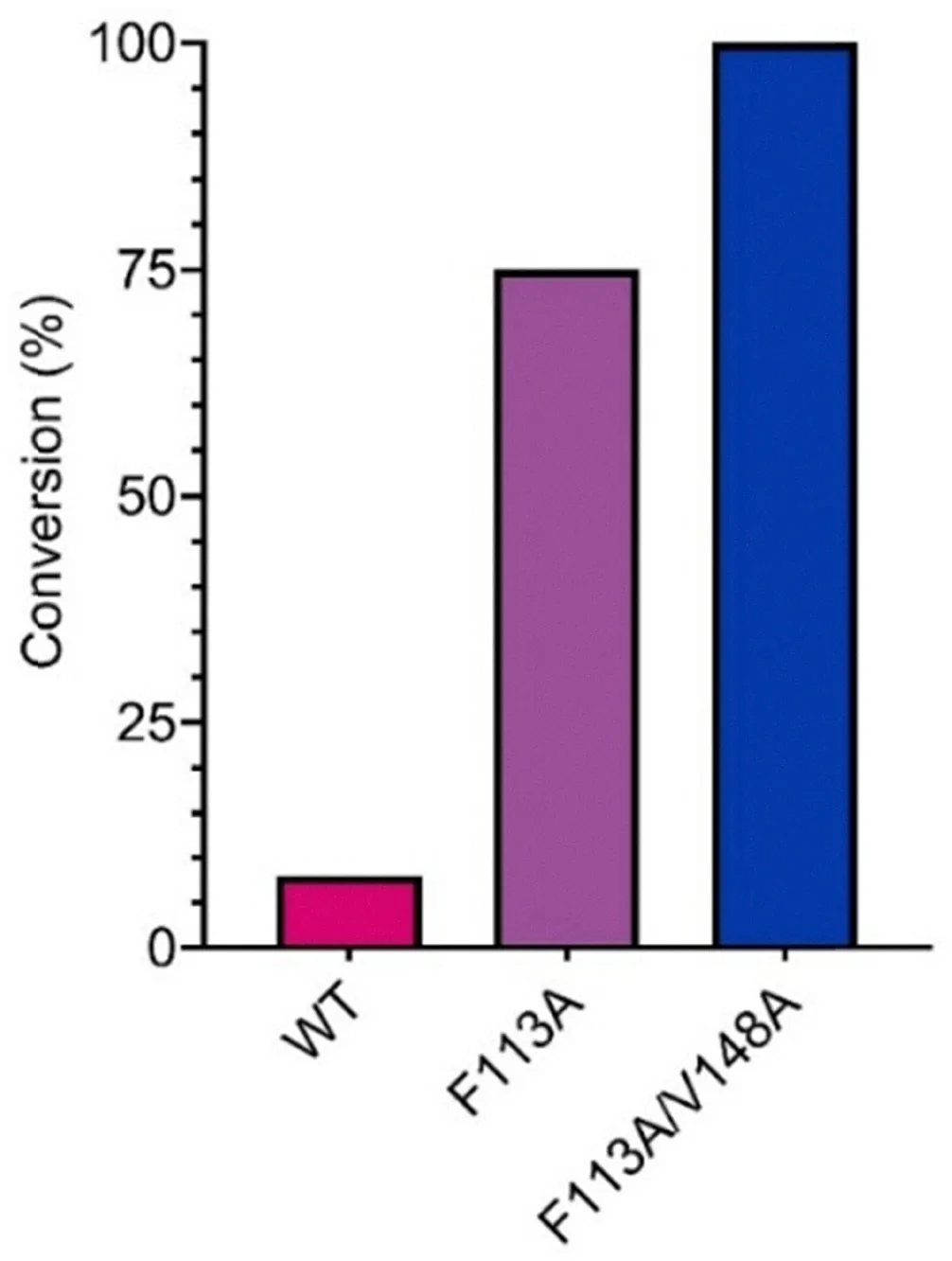
RTA-223 variant activity for the deamination of (*R*)-12 to form 13. The F113A/V148A double mutant achieved 100% conversion of (*R*)-12 and had the first detectable activity towards the formation of (*R*)-12 from precursor 13. Conditions: 5 mg mL^−1^ purified RTA-223, 5 mM sitagliptin (12), 200 mM sodium pyruvate and 100 µM PLP in 100 mM triethanolamine buffer (pH 7.5) heated at 30 °C for 24 hours.

RTA-223 was first screened against 13. The reaction was run using 5 mg mL^−1^ purified RTA-223, 5 mM ketone, 500 mM IPA, and 100 µM PLP in 5 vol% DMSO, 100 mM triethanolamine buffer (pH 7.5) at 30 °C for 24 hours, but no conversion to product was observed. Similarly, when d-alanine was used as the amine donor, no reaction took place. Activity of RTA-223 was then tested on two truncated substrates 14 and 15. Compound 14 was determined to be unsuitable as a substrate for screening due to apparent degradation in the reaction mixture. For compound 15, however, over 80% conversion of the ketone was obtained, which, by comparison with the 3% conversion observed for the starting ATA-117 construct, highlighted RTA-223 as a promising template under similar conditions, differing only by substrate loading and enzyme concentration (ATA-117 was tested at 10 mg mL^−1^ enzyme and 7.2 mM 15, whilst RTA-223 was tested at 5 mg mL^−1^ enzyme and 5 mM 15). Rather than embarking on a substrate-walking approach using substrate 15, we therefore decided to screen for the reverse deamination reaction on the fully elaborated substrate (*i.e.*(*R*)-12 to 13). We were pleased to observe an 8% conversion in 24 hours using purified enzyme (Fig. 3). We therefore elected to adopt the reverse screen for our initial rounds of evolution.

### Directed evolution of RTA-223

The crystal structure of ATA-117 was overlaid on the predicted structure of RTA-223 (Fig. S3 and S4) to help identify target residues for mutagenesis to potentially unlock activity for sitagliptin formation. One noticeable candidate near the entrance of the active site of RTA-223 was F113, which we hypothesised might inhibit bulky substrates from entering. For this reason, the F113A mutation was made to see if further improvements in activity could be obtained. Pleasingly, this rational mutation gave an eightfold increase in activity, achieving 64% conversion of (*R*)-12 to 13 in 24 hours. Despite the improvement, forwards amination activity was still not observed. Encouragingly, however, the F113A variant was not able to deaminate the (*S*)-12 enantiomer, demonstrating that (*R*)-selectivity had not been compromised by the F113A mutation. Notably, the conserved F113 position has been highlighted in the engineering of two other (*R*)-selective TAs, with F113T/L mutations enabling improved substrate access to the active site in candidates from *Capronia epimyces* (*Ce*TA) and *Gibberella zeae*, respectively.^15,25^

**Fig. 4:**
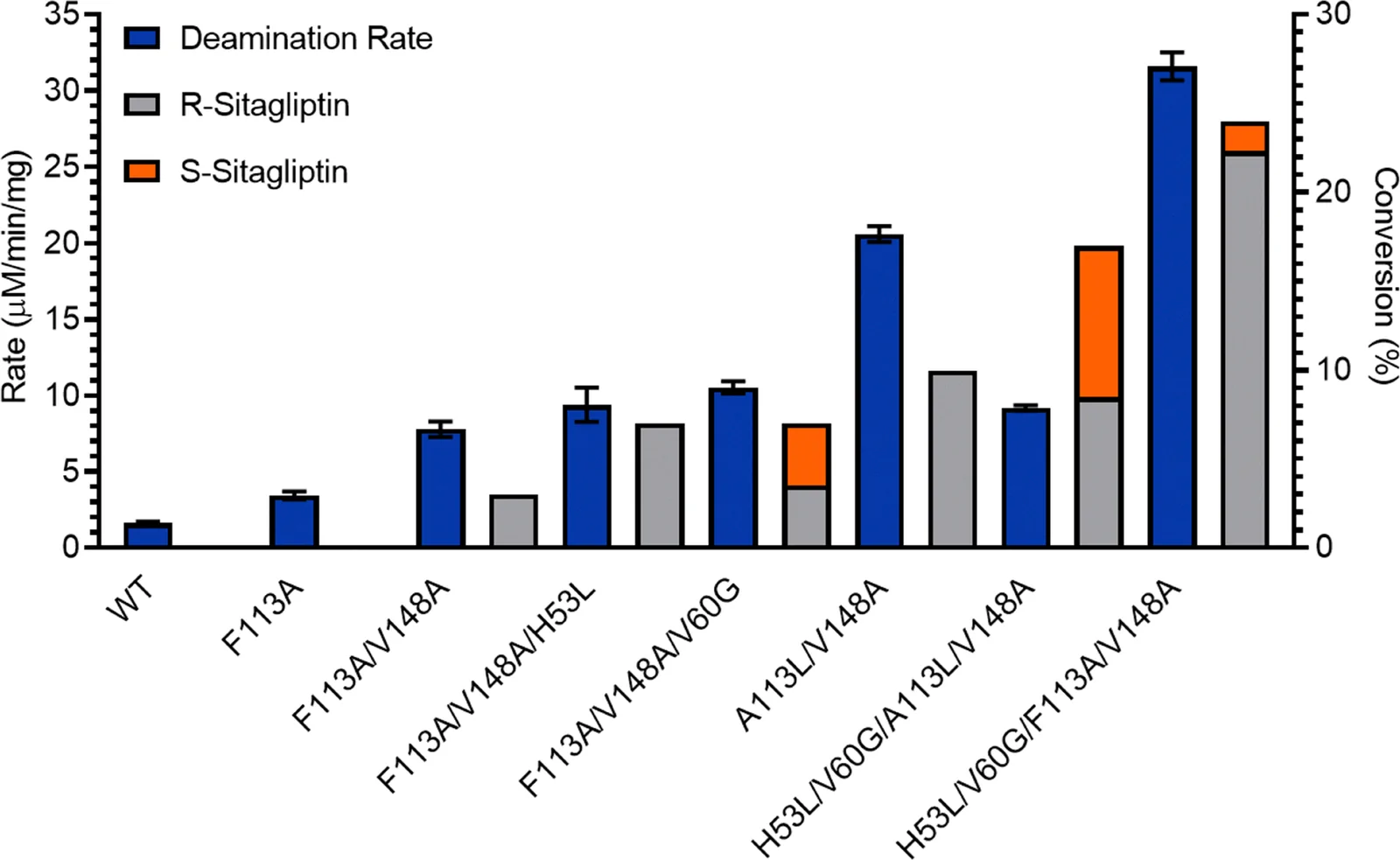
Summary of selected RTA-223 variants and their activity towards sitagliptin transamination. For each variant, the rate of deamination of (*R*)-sitagliptin ((*R*)-12) is shown in blue (scaled on the left-hand axis) and plotted alongside data for the conversion of *pro*-sitagliptin (13) into the corresponding (*R*)-12 (grey) or (*S*)-12 (orange) amine (scaled on the right-hand axis).

**Fig. 5:**
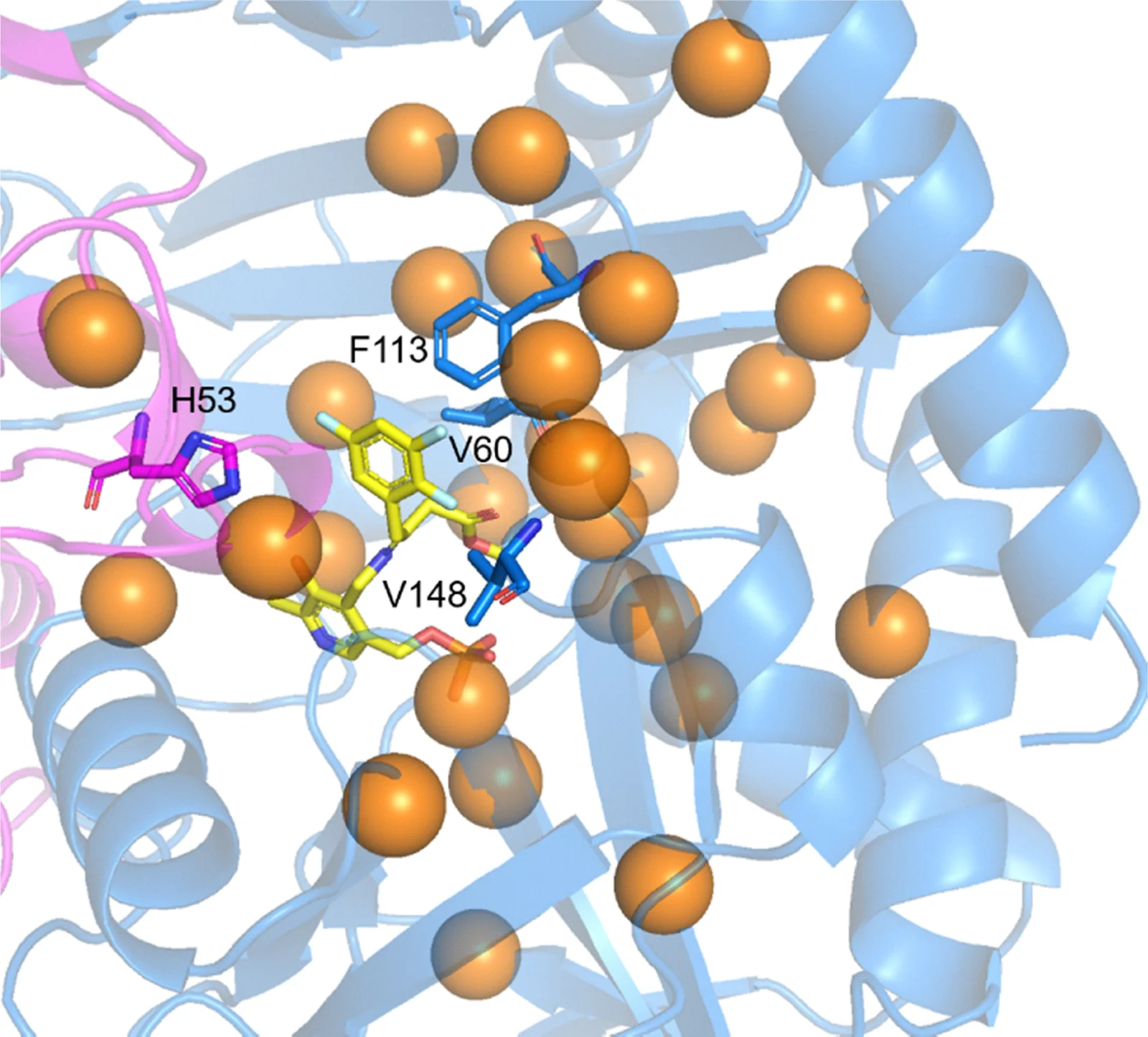
Predicted structure of the RTA-223 active site.^35^ Residues shown as sticks are the sites of the mutations found to be beneficial, whilst the orange spheres indicate the positions of all the other residues targeted during the evolution. A PLP-14 adduct docked into the structure is shown in yellow, and the pink and blue chains indicate the different units of the transaminase dimer.

RTA-223 F113A was then taken forwards for two rounds of site saturation mutagenesis (18 positions in Round 1, and then 24 positions in Round 2, sites shown in Table S.1) with the results from the best candidates summarised in Fig. 3 and 4. The positions of the most significant sites are highlighted in the structure in Fig. 5, which depicts WT RTA-223 with the PLP-14 intermediate adduct bound. The first round of evolution focused on the active site in which one beneficial mutation was found (F113A/V148A) which increased deamination from 64% to full conversion under the same conditions. The F113A/V148A variant also generated the first detectable amination activity, with a 3% conversion to (*R*)-12 using d-alanine as the amine donor. From this point onwards, amination activity was considered substantial enough to warrant screening in the forwards direction using HPLC.

The second round of evolution targeted 24 residues near the active site and at the dimer interface, using F113A/V148A as the parent. Two additional beneficial mutations were introduced to F113A/V148A: H53L and V60G, which both roughly doubled conversion of 13 to 12 relative to F113A (albeit with a loss in stereoselectivity for V60G). Re-introducing position 113 in the site saturation mutagenesis screening also proved helpful, as A113L/V148A saw the conversion to (*R*)-12 increase to 10%. Checking the structural alignment of RTA-223 and ATA-117 (Fig. S4) reveals that site H53 and V60 from RTA-223 align with sites H62 and V69 of ATA-117, respectively, both of which were influential in the evolution campaign. Furthermore, mutants of H53, V60, and V148 were also shown to increase activity in other (*R*)-TA candidates (albeit on smaller substrates).^15,25^

Gene shuffling then generated two improved variants: H53L/V60G/A113L/V148A, which increased the conversion of 13 to 12 to 17%. However, the most effective combination was H53L/V60G/F113A/V148A, which gave 24% conversion of 13 to 12 with d-alanine as an amine donor, and an e.r. of 93 : 7 towards the (*R*)-enantiomer. Switching to 500 mM IPA as the amine donor saw the conversion drop by approximately 50%, but this still provides a good starting basis for future rounds of evolution to develop an industrial biocatalyst.

### Kinetic investigation of RTA-223

**Scheme 2:**
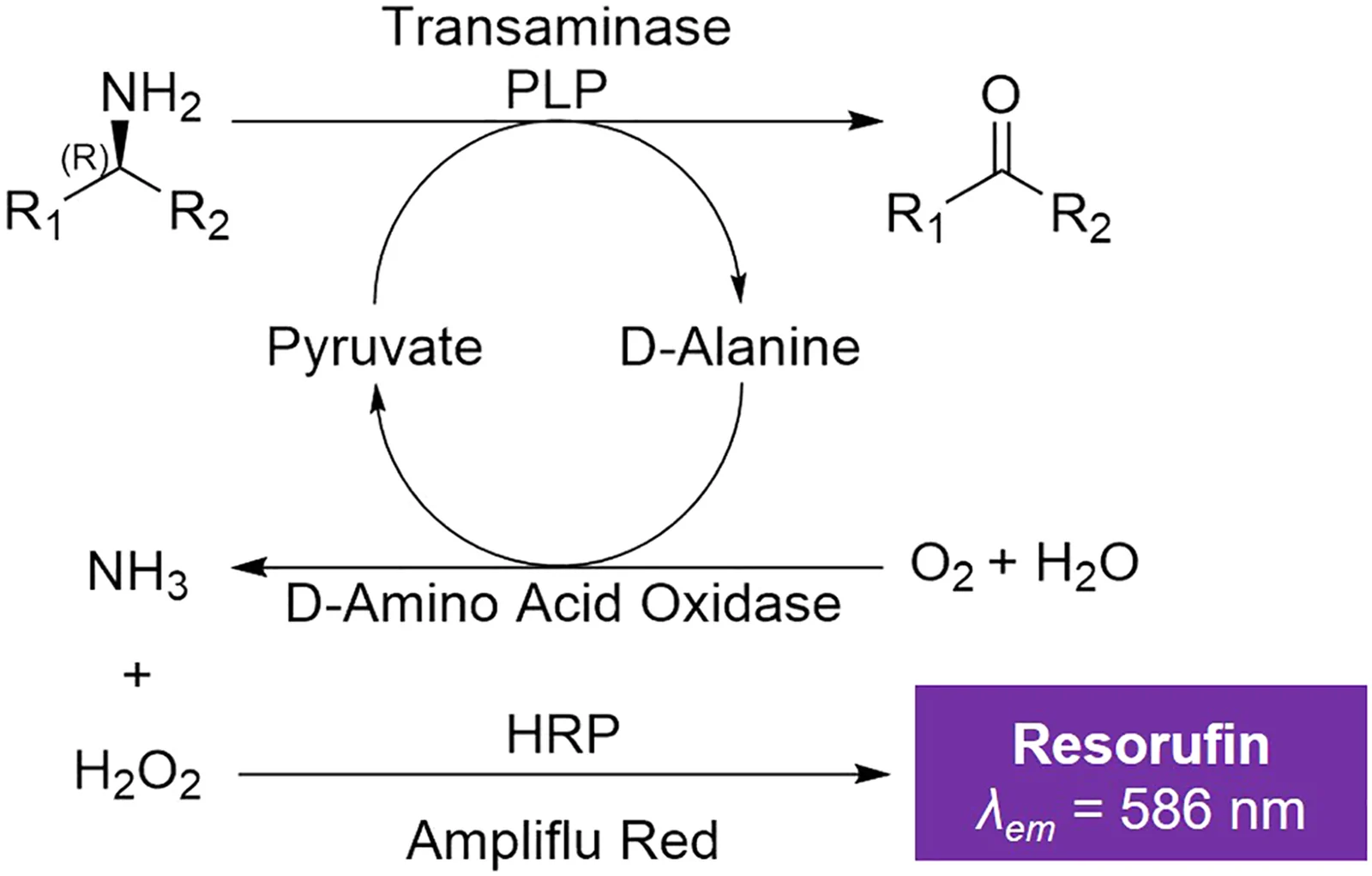
Coupled assay to follow transaminase deamination of amines.^34^

After using conversion data as the selection method to drive the directed evolution of RTA-223, we were interested to retrospectively analyse how the kinetic performance of the candidates evolved too. A fluorometric assay (Scheme 2) was therefore identified to enable determination of the early-stage rates for the deamination reactions ((*R*)-12 to 13).^34^

Using purified enzyme to study initial rates we observed that, in most cases, increases in deamination rate accompanied increases in conversion for the corresponding amination reaction (13 to (*R*)-12) (Fig. 4). The largest outlier to this trend appears to be the H53L/V60G/A113L/V148A mutant; this difference may be related to the loss of stereoselectivity (Fig. 4), as the kinetic assay depends strictly on the production of d-alanine. From these experiments, we concluded that the coupled kinetic assay is a robust method to inform directed evolution studies, and could potentially be used as the selection method in more high-throughput campaigns (such as those employing microfluidic technologies).

## Conclusion

In this study we report the discovery, recombinant expression, and engineering of a novel transaminase, RTA-223, which has (*R*)-selective amination activity for a range of sterically hindered ketone substrates. As a WT enzyme, RTA-223 can utilise IPA or d-alanine as an amine donor. WT RTA-223 also has limited activity for deamination of (*R*)-sitagliptin, a feature that we were able to exploit for unlocking activity towards *pro*-sitagliptin after only two mutations. A further round of directed evolution gave a quadruple mutant that can perform 24% conversion of pro-sitagliptin to (*R*)-sitagliptin (93 : 7 e.r.). These features highlight RTA-223 as a good candidate for other evolution campaigns on bulky substrates or as a starting point for exploring similar sequences. The implemented coupled peroxide assay verified that increasing deamination rate across the various candidates is accompanied by improvements in corresponding amination activity. Given its high sensitivity and easy implementation, the assay could prove valuable for future directed evolution campaigns of (*R*)-TAs, especially where chromatographic methods act as a bottleneck for screening.

## Conflicts of interest

There are no conflicts to declare.

## Supplementary Material

CB-OLF-D6CB00180G-s001

## Data Availability

The data supporting this article (UPLC and GC traces, kinetic plots, bioinformatic analyses) have been included as part of the supplementary information (SI). Supplementary information is available. See DOI: https://doi.org/10.1039/d6cb00180g. Any additional raw data can be made available by the corresponding author upon request.
