# Supplementary material for: Discovery and evolution of an (R)-selective transaminase for production of sitagliptin
Source: RSC Chem Biol. 2026 Aug 20;7(10):2053–8. doi: 10.1039/d6cb00180g (PMC13491446; doi:10.1039/d6cb00180g)
Supplement: Supplementary file 1 [file CB-007-D6CB00180G-s001.pdf]

Supplementary Information (SI) for RSC Chemical Biology.
This journal is © The Royal Society of Chemistry 2026

Electronic Supplementary Information

Discovery and evolution of an (*R*)-selective transaminase for production of sitagliptin

Matthew Treadell, Gareth R. E. Surman, Grayson J. Ford, Christopher W. Otun, Shiny Joseph Srinivasan, James Galman, Maria Bawn, Akash Pandya, Craig J. Markin, Anthony P. Green, Jack S. Rowbotham*

*Author for correspondence: jack.rowbotham@manchester.ac.uk

Contents

1. Methodology 3

1.1 Materials and reagents 3

1.2 General protocol for protein expression 3

1.3 General protocol for protein purification 3

1.4 General protocol for transaminase biotransformations 4

1.5 UPLC analysis 5

1.6 Chiral GC analysis 6

1.7 Point mutations by overlap extension PCR 6

1.8 Library construction 6

1.9 Gene shuffling by overlap extension PCR 7

1.10 Library growth and screening 7

1.11 Coupled assay to detect transaminase activity 7

1.12 Phylogenetic tree generation 8

2. RTA-223 sequences 8

3. Bioinformatics of transaminase sequences 10

4. RTA-223 structure and SDS-PAGE gel 11

5. Chromatograms from biotransformations 12

5.1 Data to determine reaction conversion in substrate screening 12

5.2 Data to determine enantiomeric ratio 16

6. Sites targeted during evolution 18

7. Fluorescence assay to determine transaminase rate 19

8. Primers 21

1. Methodology

1.1 Materials and reagents

All commercial materials were used as purchased without purification, unless stated otherwise. Materials used for protein expression and purification included: terrific broth (TB), lauryl broth (LB), kanamycin, isopropyl β-D-1-thiogalactopyranoside (IPTG) and HEPES obtained from Formedium, sodium chloride from Fischer; imidazole from Fluorochem and pyridoxal 5-phosphate (PLP) from Merck.

Compounds used in the substrate scope include: triethanolamine, ammonium formate, acetophenone (**1**), 3-fluoroacetophenone (**3**), 4-hydroxyacetophenone (**5**), 2-acetonapththalin (**9**) and 2-phenylcyclohexanone (**10**) from Merck; 4-nitroacetophenone (**2**), 5-acetyl-2-chloropyridine (**4**), 3-acetylpyridine (**6**), 3,5-dimethoxyacetophenone (**8**), 1,3-diphenylpropan-2-one (**11**) and (*S*)- and (*R*)-α-methylbenzylamine from Fluorochem; 2-acetylpyrazine (**7**) and D- and L-alanine from Alfa Aesar.

Additional materials used in directed evolution campaign include: (*R*)-sitagliptin (**(*R*)-12**), pro-sitagliptin (**13**), 1-[3-(trifluoromethyl)-5,6-dihydro-[1,2,4] triazole[4,3-a]pyrazin-7(8H-yl]-1,3-butanedione (**15**) from Biosynth; methyl 3-oxo-4-(2,4,-trifluorophenyl) butanoate (**14**) from Fluorochem; isopropylamine hydrochloride, polymyxin B from Apollo Scientific; lysozyme, sodium pyruvate, ampliflu red, resorufin and (*S*)-sitagliptin (**(*S*)-12**) from Merck.

All reactions and stock solutions were prepared using MilliQ ultrapure water with a resistivity of 18.2 MΩ.cm at 25 °C.

1.2 General protocol for protein expression

All molecular biology work was carried out under sterile conditions with autoclaved media and plasticware. *E. coli* BL21 (DE3) was first transformed with the necessary plasmid (such as pET28a-RTA-223) and single colonies were grown overnight on agar plates containing kanamycin. A single colony was picked and added to 5 mL of LB medium supplemented with 50 μg/mL of kanamycin, and incubated at 37°C for overnight. 1 mL of the inoculum was added to 100 mL TB medium with 50 μg/mL kanamycin and shaken at 200 rpm at 37 °C until the optical density at 600 nm (OD_{600}) reached 0.7 to 0.8. Protein expression was then induced with 0.1 mM IPTG, and the culture temperature was lowered to 25 °C for 20 hours.

1.3 General protocol for protein purification

The bacterial cells were harvested by centrifugation at 8085 × g, 4 °C for 20 min. The supernatant was removed before the cells were resuspended in 50 mM HEPES (pH 7.5) with 200 mM NaCl, 100 μM PLP, and 10 mM imidazole. Cell membranes were disrupted by

ultrasonication. The cell debris was then removed by centrifugation at 8085 × g, 4 °C for 20 minutes. The recombinant RTA-223 was purified with a Ni^{2+} affinity column. Purification was carried out using a buffer solution of 50 mM HEPES (pH 7.5) with 200 mM NaCl, 100 μM PLP, washing with 50 mM imidazole and eluting with 250 mM imidazole.

Desalting and buffer exchange were carried out on the eluted fraction using a PD 10 column, exchanging the buffer to a 50 mM HEPES (pH 7.5) solution containing 100 μM PLP. Enzyme samples were concentrated using 30 kDa Vivaspin columns.

Protein concentration was determined using a NanoDrop Microvolume Spectrophotometer assuming a protein extinction coefficient of 49515 M^{-1} cm^{-1} and a molecular weight of 38.7 kDa.

1.4 General protocol for transaminase biotransformations

Amination reactions: biotransformations were carried out on a 100 μL scale in a 1.5 mL Eppendorf microcentrifuge tube, using 5 mg/mL RTA-223, 5 mM ketone, 500 mM amine donor and 100 μM PLP in 100 mM triethanolamine buffer (pH 7.5) at 30 °C and 800 rpm for 24 hours. Amine donors used were either D-alanine (**2-11**, **13**) or $^{i}PrNH_2$ (**1**, **13**-**15**). Reactions were quenched by the addition of an equal volume of acetonitrile containing 0.2 vol.% formic acid. Reactions were analysed using UPLC and GC, according to the conditions in Section 1.5.

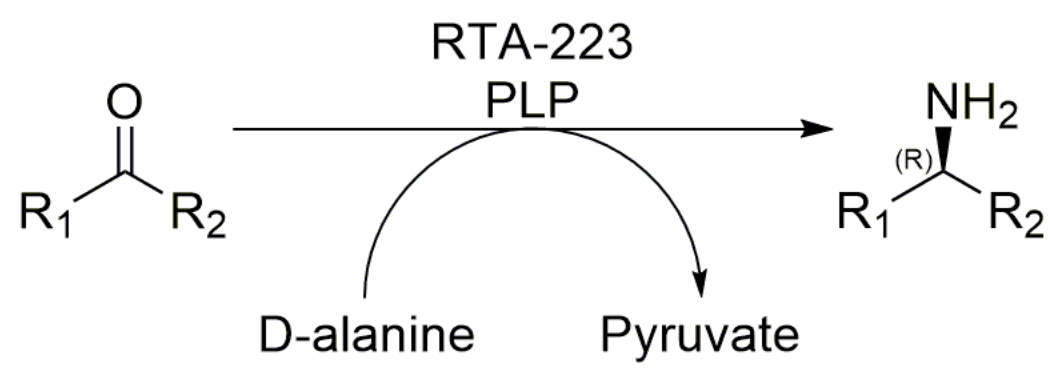

Scheme S. 1: Generic reaction scheme for studying RTA-223 amination activity

Deamination reactions: biotransformations were carried out on a 100 μL scale in a 1.5 mL Eppendorf microcentrifuge tube, using 5 mg/mL RTA-223, 5 mM sitagliptin (**12**), 200 mM sodium pyruvate and 100 μM PLP in 100 mM triethanolamine buffer (pH 7.5) at 30 °C and 800 rpm for 24 hours. Reactions were quenched by the addition of an equal volume of acetonitrile containing 0.2 vol.% formic acid. Reactions were analysed using UPLC, according to the conditions in Section 1.5.

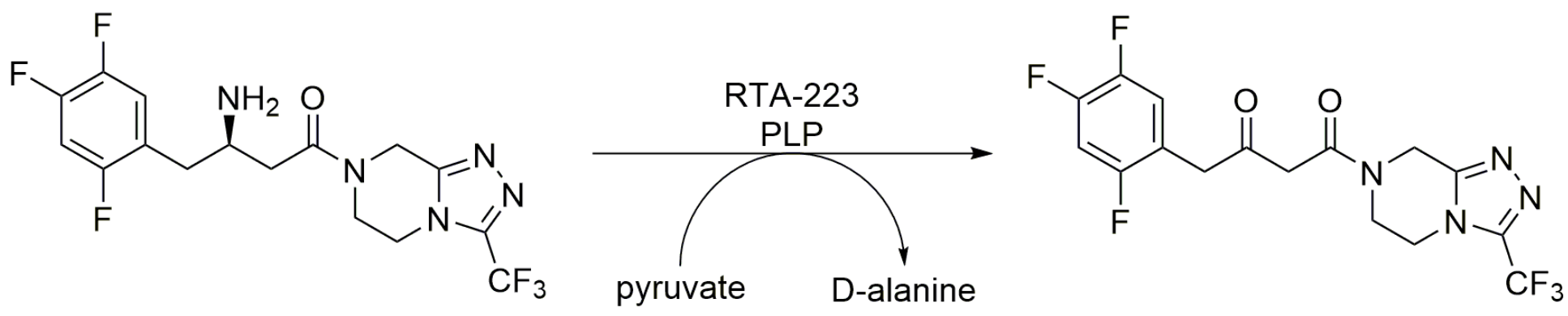

Scheme S. 2: Deamination of sitagliptin by RTA-223

1.5 UPLC analysis

UPLC for exploring transaminase substrate scope

UPLC analysis was carried out using an Agilent 1290 Infinity II.

For compounds **2**, **4-6** and **9-11**, the UPLC was equipped with an Agilent InifintiyLab poroshell 120 EC-C18 column (2.1 x 50 mm, 1.9 μm) using a H_2O/MeCN eluent system containing 0.1% formic acid, using a 5% to 95% MeCN gradient, with 0.5 mL/min rate at 25 °C and, a 2 μL sample injection.

For compounds **3**, **7** and **8**, the UPLC was equipped with an Acquity UPLC glycan BEH amide column (2.1 x 150 mm, 1.7 μm). Two mobile phases were used: A) 95:5 MeCN:H_2O, and B) 5:95: MeCN:H_2O. A gradient elution was then used transitioning from 100% A / 0% B to 10% A / 90% B, with 0.5 mL/min flow rate at 25 °C and, a 2 μL sample injection.

Detections for **1-11** were carried out by using UV wavelengths at 210 nm, 254 nm and 280 nm, along with mass detection in positive ion mode.

Chiral UPLC for determining enantiomeric ratio

Chiral UPLC was carried out using an Agilent 1290 Infinity II, equipped with an Agilent InifintiyLab poroshell 120 chiral-V column (4.6 x 100 mm, 2.7 μm). Separation was achieved using the following eluent system for each compound:

Compounds **2** and **6**: 70:30 acetonitrile: 20 mM ammonium formate (pH 4) 0.25 mL/min

Compound **7**: 50:50 acetonitrile: 20 mM ammonium formate (pH 4) 0.25 mL/min

Compounds **4**, **10**, and **12**: 100% MeOH containing 0.2% ammonium formate at 0.5 mL/min

UPLC for screening transaminase activity towards sitagliptin

UPLC analysis was carried out using an Agilent 1290 Infinity II UPLC equipped with an Agilent InifintiyLab poroshell 120 EC-C18 column (2.1 x 50 mm, 1.9 μm) using a H_2O/MeCN eluent system containing 0.1 vol.% formic acid, using a 30 vol.% to 95 vol.% MeCN gradient, with a flow rate of 1.25 mL/min.

Retention times: Sitagliptin (**12**) = 0.18 min; *pro*-sitagliptin (**13**) = 0.53 min

Conversions were determined using the peak area at 268 nm.

1.6 Chiral GC analysis

Derivatisation method for chiral GC analysis

For Compound **1**, 100 µL of reaction was quenched by the addition of 5 M NaOH (10 µL) and extracted into EtOAc (200 µL). The organic layer was dried over anhydrous $MgSO_4$, mixed with 1.4 M *N*-methyl-bis(trifluoroacetamide) (MBTFA) in DCM (200 µL), and heated at 60 °C for 1 hour. The resulting mixture was transferred to a glass vial for chiral GC-FID analysis.

Chiral GC-FID was carried out on an Agilent 6850A (G2630B) gas chromatography system equipped with an Agilent 6850 autosampler. The column and chromatographic conditions are stated below:

Column: MilliporeSigma Supelco β-DEX 325 (30 m x 0.25 mm x 0.25 µm)
Carrier: 1.2 mL/min He (constant flow)
Inlet temperature: 200 °C
Injection gases: Split with split flow 120 mL min^{-1}, split ratio 100:1
Injection volume: 1 µL
Detection: Flame ionisation detection (FID)
Detector temperature: 300 °C
Detection gases: H_2 (40 mL/min), air (450 mL/min), makeup He (45 mL/min)
Oven heating profile: 0 to 80 mins ramp from 50 °C to 130 °C at 1 °C/min

1.7 Point mutations by overlap extension PCR

Point mutations were created using primers encoding the desired residue, using overlap extension PCR to construct the mutated gene. The linear gene was digested using NdeI and XhoI restriction enzymes and ligated into pET-28a using T4 DNA ligase.

1.8 Library construction

24 positions were individually randomised using primers with NNK degenerate codons. DNA libraries were constructed using overlap extension PCR. The linear library genes were digested using NdeI and XhoI restriction enzymes and ligated into pET-28a using T4 DNA ligase.

1.9 Gene shuffling by overlap extension PCR

Beneficial activity found from site saturation screening was combined by DNA shuffling of fragments generated by overlap extension PCR. Primers were designed that encoded either the parent amino acid or the identified mutation. These primers were used to generate short fragments, which were mixed appropriately in overlap extension PCR to generate genes containing all possible combinations of mutations. Genes were cloned into pET-28A using T4 DNA ligase

1.10 Library growth and screening

DNA Libraries were transformed into *E. coli* BL21 (DE3) competent cells. From the transformation, individual colonies were transferred to an individual well of a 96-well plate containing 180 µL LB with 500 µg/mL of kanamycin, sealed with breath-easy sealing membranes and incubated overnight at 30 °C with 80% humidity and shaking at 850 rpm. 20 µL from each well of the overnight was transferred to the corresponding well of 96 deep well plate contain 480 µL of auto-induction terrific broth with 50 µg/mL kanamycin, sealed with breath-easy sealing membranes and grown for 20 hours at 30 °C, 80% humidity with shaking at 850 rpm.

The bacterial cells were harvested by centrifugation at 8085 × g, 4 °C for 20 min. Supernatant was removed before cells were resuspended in 200 µL lysis buffer (100 mM triethanolamine buffer at pH 7.5, containing 1 mg/mL lysozyme, 0.5 mg/mL polymyxin B and 10 µg/mL DNase I) and incubated for 30 minutes at 30 °C with shaking at 850 rpm. Cell debris was removed by centrifugation at 8085 × g, 4 °C for 20 min.

Conversion data was collected using 80 µL lysate supernatant with 5 mM sitagliptin (**12**) and 200 mM pyruvate with 100 µM PLP, in 100 mM Triethanolamine buffer (pH 7.5), with a total reaction volume of 100 µL in 96-well plates and incubated at 30 °C for 24 hours, sealed using foil heat seal. Reactions were quenched using 100 µL acetonitrile containing 0.2 vol.% formic acid, and insoluble protein was removed by centrifugation at 8085 × g, 4 °C. Reaction mixtures were analysed by UPLC, as described in Section 1.5.

1.11 Coupled assay to detect transaminase activity

Rates of reaction were determined using 5 mM sitagliptin (12), 200 mM sodium pyruvate, 100 µM PLP, 100 µM Amplex Red, 10 units D-amino acid oxidase and 10 units horseradish peroxygenase in 100 mM triethanolamine buffer (pH 7.5) at 30 °C. For WT RTA-223 and variants up to the triple mutations, 2.5 mg/mL enzyme was used. For the quadruple mutants 1 mg/mL enzyme was used.

Reactions were run in a Black Corning 384-well flat-bottom plate (50 µL reaction scale) in a BMG Labtech CLARIOstar. For detection of resorufin: excitation at 570 nm, emission at 584 nm.

1.12 Phylogenetic tree generation

Sequences were gathered from the UniProt database based on a BLAST search of the *Cs*TA amino acid sequence using standard settings, along with known patented sequences. Sequences with highly divergent regions suggesting errors in annotation were removed heuristically. The remaining sequences were then aligned using MAFFT in Unipro UGENE (v51.0), and phylogeny inferred using IQ-TREE 3 (v3.1.3) with model finder identifying LG+I+G4 as the best fit model according to both the Akaike Information Criterion and Bayesian Information Criterion. The tree is unrooted, and the ultrafast bootstrap values presented as a % based on 1000 resamplings.

2. RTA-223 sequences

RTA-223

MASMDKLFAGYKSRLAALKASDNPLSQGVAWVEGEIVPLSQARIPLLDQGFLHSDLTYDVPA
VWDGRFFRLDDHLSRLEKSCSKLRLKLPFPREEVKRLLIDMVTKSGIRDVFVELIVTRGLKG
VREAKNPEDLVNTLYMFIQPYIWVMEPEVQRVGGSAVIARTVRRTPPGSMDPTVKNLQWG
DLVRAMLEASDRGAAYPFLTDGDANITEGSGFNIVLVKDGVIYTPDRGVLEGVTRKSVFDVA
KANGIEVRLEVVPVELAYQADEIFMCTTAGGIMPITSLDGQPVKDGKIGPVTQKIWDDYWAL
HYDPAYSFEIDYEGVGKNGSNGMNGIH*

F113A

MASMDKLFAGYKSRLAALKASDNPLSQGVAWVEGEIVPLSQARIPLLDQGFLHSDLTYDVPA
VWDGRFFRLDDHLSRLEKSCSKLRLKLPFPREEVKRLLIDMVTKSGIRDVAVELIVTRGLKG
VREAKNPEDLVNTLYMFIQPYIWVMEPEVQRVGGSAVIARTVRRTPPGSMDPTVKNLQWG
DLVRAMLEASDRGAAYPFLTDGDANITEGSGFNIVLVKDGVIYTPDRGVLEGVTRKSVFDVA
KANGIEVRLEVVPVELAYQADEIFMCTTAGGIMPITSLDGQPVKDGKIGPVTQKIWDDYWAL
HYDPAYSFEIDYEGVGKNGSNGMNGIH*

F113A/V148A

MASMDKLFAGYKSRLAALKASDNPLSQGVAWVEGEIVPLSQARIPLLDQGFLHSDLTYDVPA
VWDGRFFRLDDHLSRLEKSCSKLRLKLPFPREEVKRLLIDMVTKSGIRDVAVELIVTRGLKG
VREAKNPEDLVNTLYMFIQPYIWAMEPEVQRVGGSAVIARTVRRTPPGSMDPTVKNLQWGD
LVRAMLEASDRGAAYPFLTDGDANITEGSGFNIVLVKDGVIYTPDRGVLEGVTRKSVFDVAK
ANGIEVRLEVVPVELAYQADEIFMCTTAGGIMPITSLDGQPVKDGKIGPVTQKIWDDYWALH
YDPAYSFEIDYEGVGKNGSNGMNGIH*

H53L/F113A/V148A

MASMDKLFAGYKSRLAALKASDNPLSQGVAWVEGEIVPLSQARIPLLDQGFLLSDLTYDVPA
VWDGRFFRLDDHLSRLEKSCSKLRLKLPFPREEVKRLLIDMVTKSGIRDVAVELIVTRGLKG
VREAKNPEDLVNTLYMFIQPYIWAMEPEVQRVGGSAVIARTVRRTPPGSMDPTVKNLQWGD
LVRAMLEASDRGAAYPFLTDGDANITEGSGFNIVLVKDGVIYTPDRGVLEGVTRKSVFDVAK
ANGIEVRLEVVPVELAYQADEIFMCTTAGGIMPITSLDGQPVKDGKIGPVTQKIWDDYWALH
YDPAYSFEIDYEGVGKNGSNGMNGIH*

V60G/F113A/V148A

MASMDKLFAGYKSRLAALKASDNPLSQGVAWVEGEIVPLSQARIPLLDQGFLHSDLTYDGP
AVWDGRFFRLDDHLSRLEKSCSKLRLKLPFPREEVKRLLIDMVTKSGIRDVAVELIVTRGLKG
VREAKNPEDLVNTLYMFIQPYIWAMEPEVQRVGGSAVIARTVRRTPPGSMDPTVKNLQWGD
LVRAMLEASDRGAAYPFLTDGDANITEGSGFNIVLVKDGVIYTPDRGVLEGVTRKSVFDVAK
ANGIEVRLEVVPVELAYQADEIFMCTTAGGIMPITSLDGQPVKDGKIGPVTQKIWDDYWALH
YDPAYSFEIDYEGVGKNGSNGMNGIH*

A113L/V148A

MASMDKLFAGYKSRLAALKASDNPLSQGVAWVEGEIVPLSQARIPLLDQGFLHSDLTYDVPA
VWDGRFFRLDDHLSRLEKSCSKLRLKLPFPREEVKRLLIDMVTKSGIRDVLVELIVTRGLKGV
REAKNPEDLVNTLYMFIQPYIWAMEPEVQRVGGSAVIARTVRRTPPGSMDPTVKNLQWGDL
VRAMLEASDRGAAYPFLTDGDANITEGSGFNIVLVKDGVIYTPDRGVLEGVTRKSVFDVAKA
NGIEVRLEVVPVELAYQADEIFMCTTAGGIMPITSLDGQPVKDGKIGPVTQKIWDDYWALHY
DPAYSFEIDYEGVGKNGSNGMNGIH*

H53L/V60G/F113A/V148A

MASMDKLFAGYKSRLAALKASDNPLSQGVAWVEGEIVPLSQARIPLLDQGFLLSDLTYDGPA
VWDGRFFRLDDHLSRLEKSCSKLRLKLPFPREEVKRLLIDMVTKSGIRDVAVELIVTRGLKG
VREAKNPEDLVNTLYMFIQPYIWAMEPEVQRVGGSAVIARTVRRTPPGSMDPTVKNLQWGD
LVRAMLEASDRGAAYPFLTDGDANITEGSGFNIVLVKDGVIYTPDRGVLEGVTRKSVFDVAK
ANGIEVRLEVVPVELAYQADEIFMCTTAGGIMPITSLDGQPVKDGKIGPVTQKIWDDYWALH
YDPAYSFEIDYEGVGKNGSNGMNGIH*

H53L/V60G/A113L/V148A

MASMDKLFAGYKSRLAALKASDNPLSQGVAWVEGEIVPLSQARIPLLDQGFLLSDLTYDGPA
VWDGRFFRLDDHLSRLEKSCSKLRLKLPFPREEVKRLLIDMVTKSGIRDVLVELIVTRGLKGV
REAKNPEDLVNTLYMFIQPYIWAMEPEVQRVGGSAVIARTVRRTPPGSMDPTVKNLQWGDL
VRAMLEASDRGAAYPFLTDGDANITEGSGFNIVLVKDGVIYTPDRGVLEGVTRKSVFDVAKA
NGIEVRLEVVPVELAYQADEIFMCTTAGGIMPITSLDGQPVKDGKIGPVTQKIWDDYWALHY
DPAYSFEIDYEGVGKNGSNGMNGIH*

3. Bioinformatics of transaminase sequences

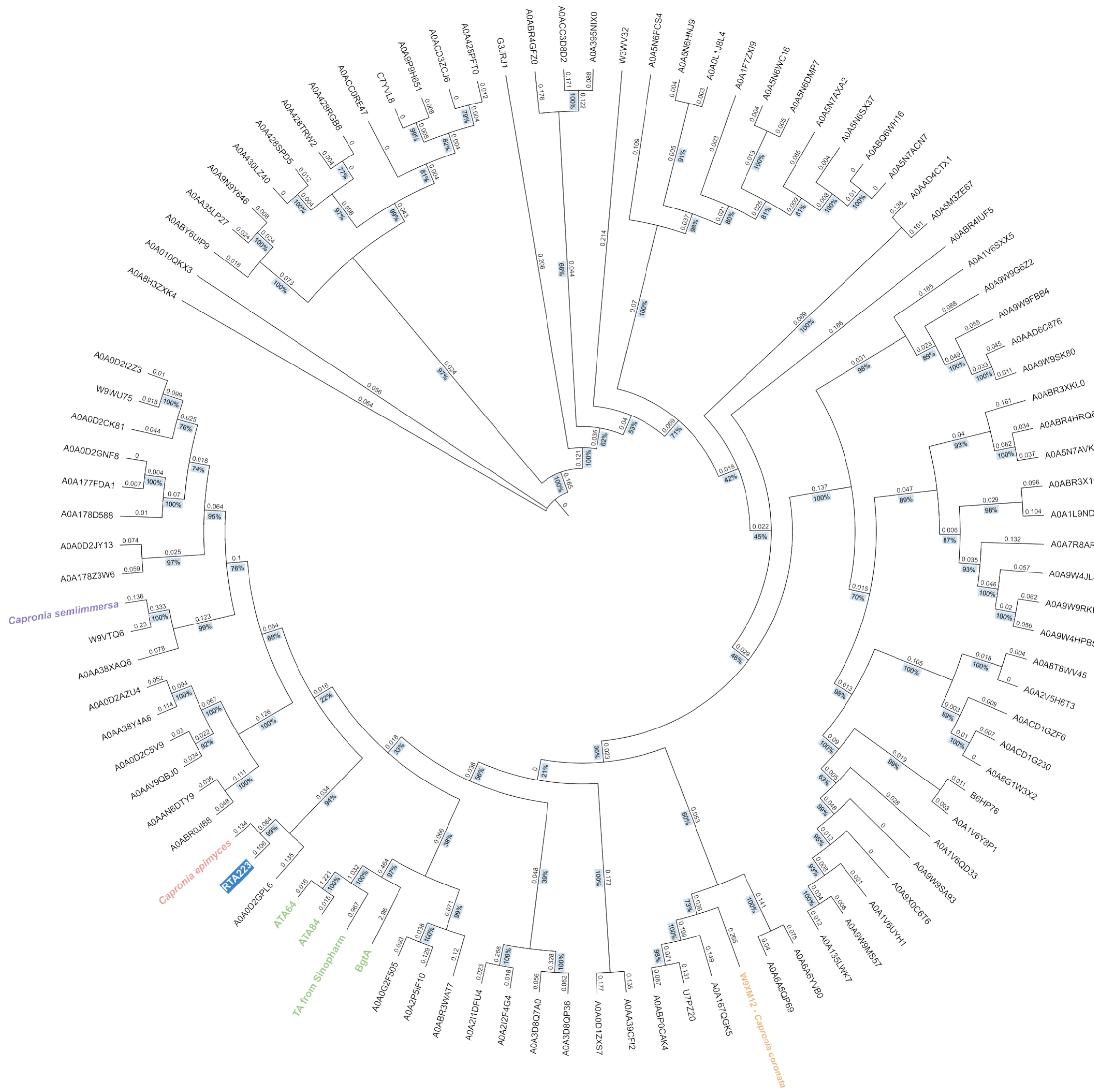

Figure S.1: Phylogeny of the sequences from the BLAST search of CsTA, with *Capronia* species highlighted. ATA-64, ATA-84, BgTA and a TA from Sinopharm Weiqida Pharmaceuticals have been included to show their similarity to RTA-223. The tree is unrooted and shown in circular format, visualised using iTOL (Version 7).

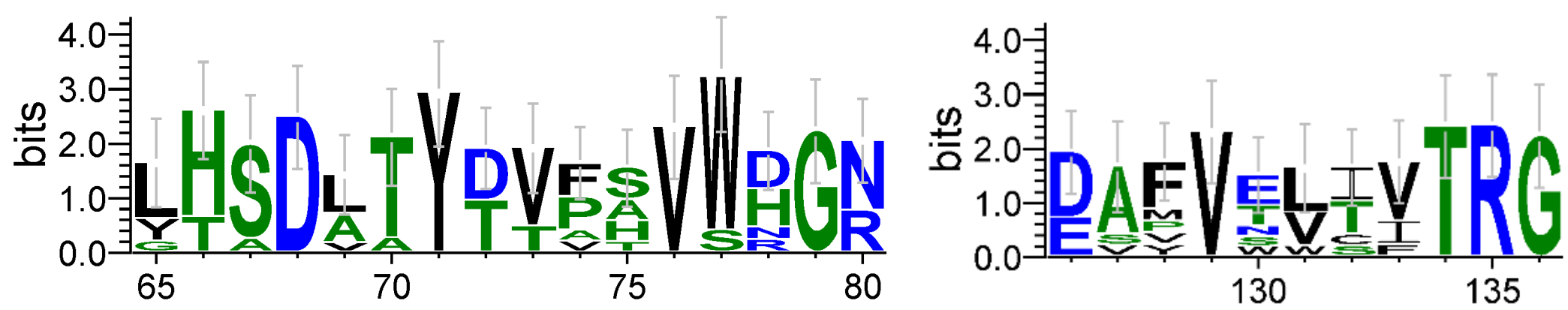

Figure S.2: Amino acid sequence logo of transaminase candidates: ATA-117, RTA-223, *Capronia semimmersa*, *Capronia epimyces*, ATA-64, ATA-84, BgTA, and a transaminase from Sinopharm Weiqida Pharmaceuticals, on which the numbering is based.

4. RTA-223 structure and SDS-PAGE gel

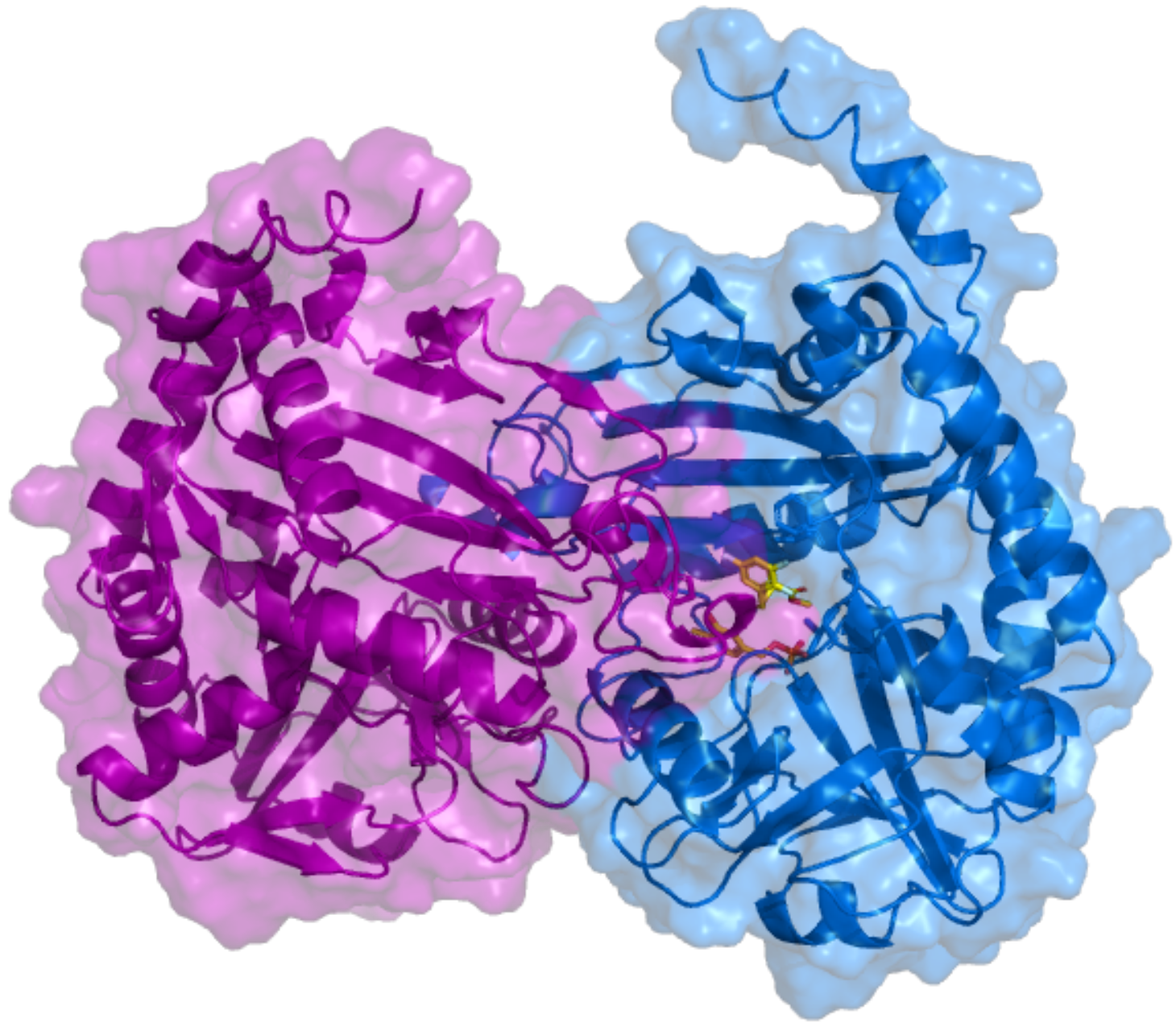

Figure S.3: Structure of RTA-223 predicted using AlphaFold v2. As expected, the model depicts RTA-223 transaminase as a dimer. pLDDT: 94.5 %

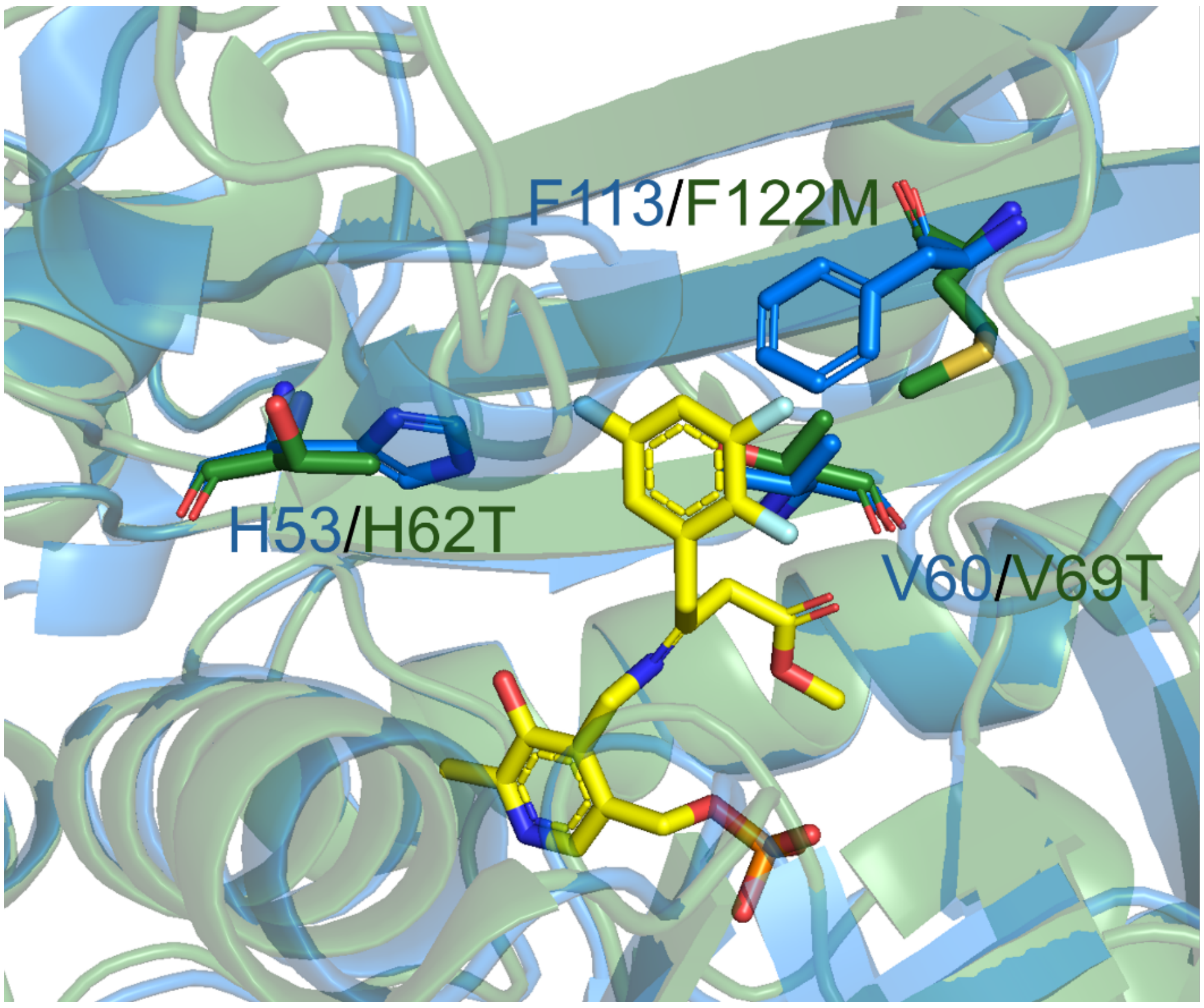

Figure S.4: Structure alignment of ATA-117 final evolved variant (green) and RTA-223 (blue). The highlighted residues show sites that were targeted in the evolution campaigns of the two candidates, and which show close structural alignment.

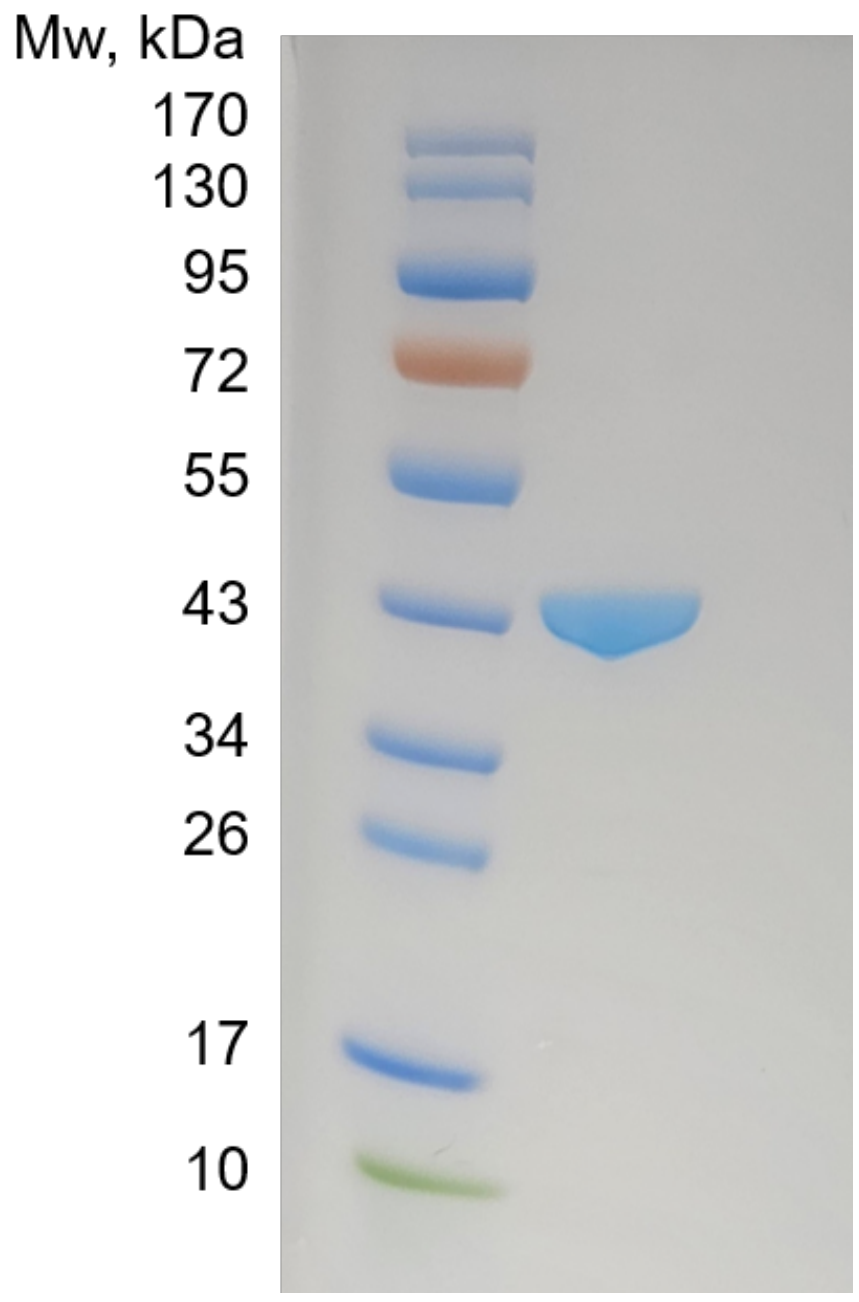

Figure S.5: SDS-PAGE gel of purified RTA-223

5. Chromatograms from biotransformations

5.1 Data to determine reaction conversion in substrate screening

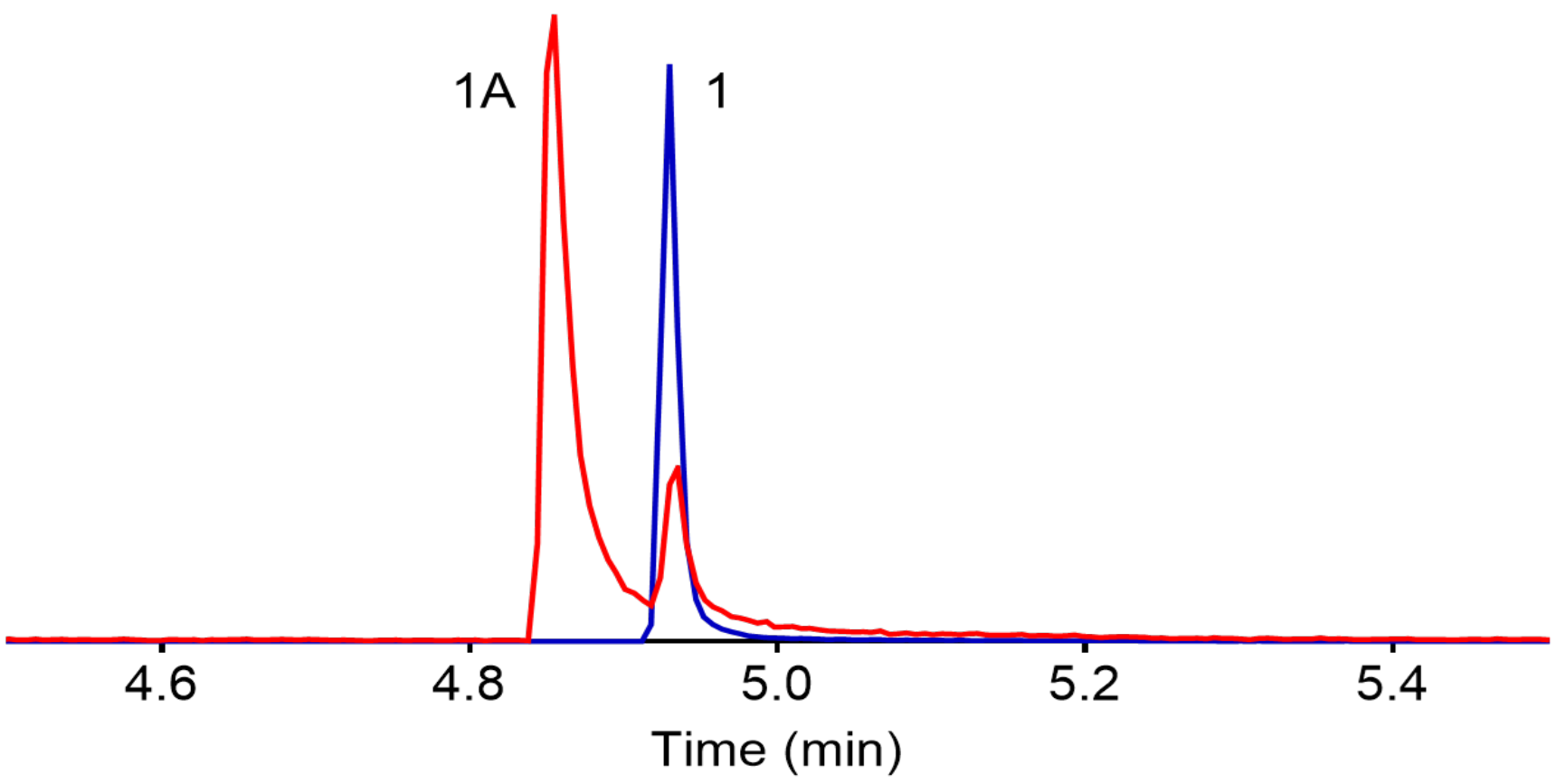

Figure S. 6: GC trace for the amination of Compound **1** by RTA-223. Red: biotransformation, Blue: Standard of **1.**

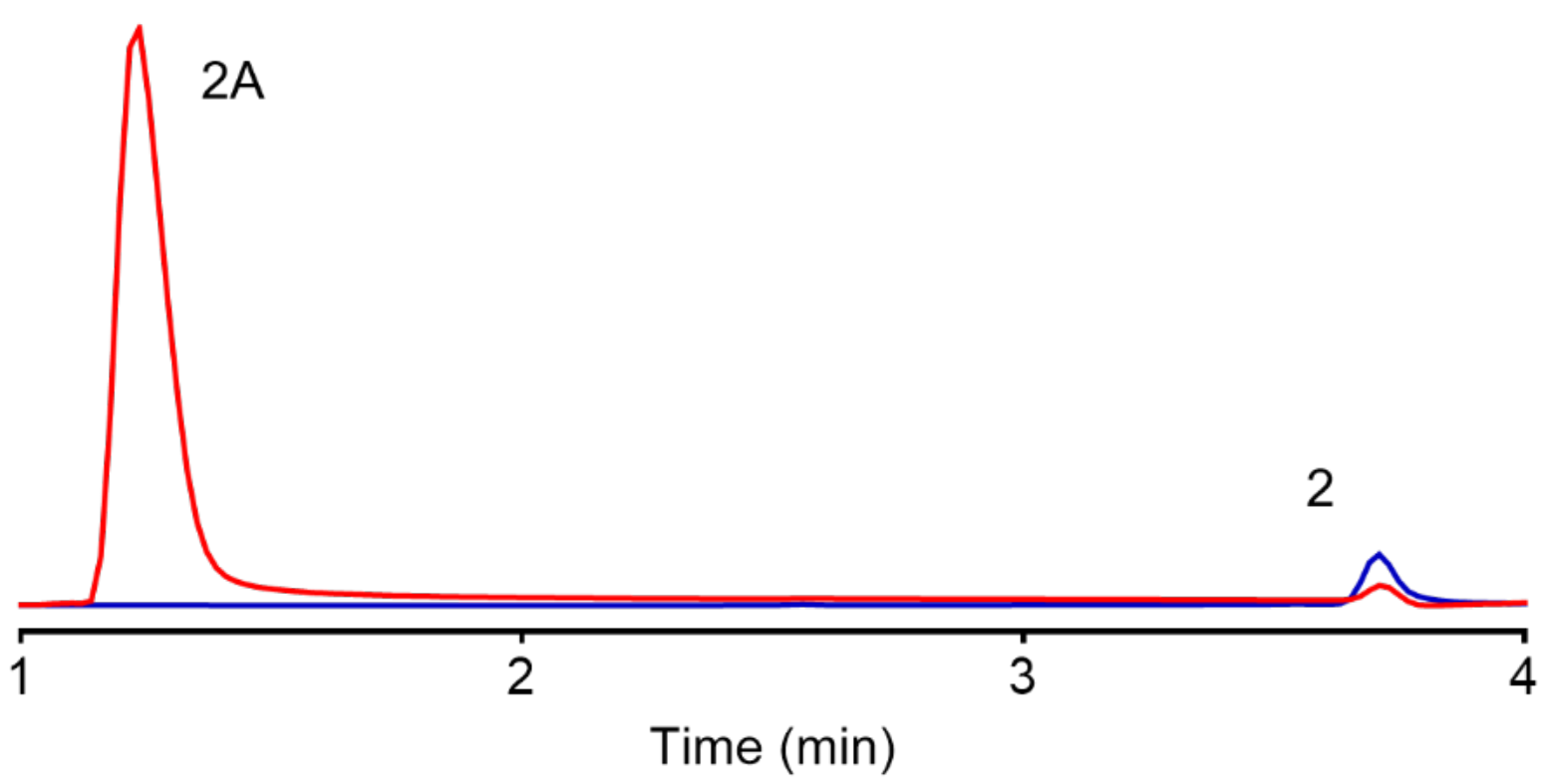

Figure S. 7: UPLC trace for the amination of Compound **2** by RTA-223. Red: biotransformation, Blue: standard of **2**.

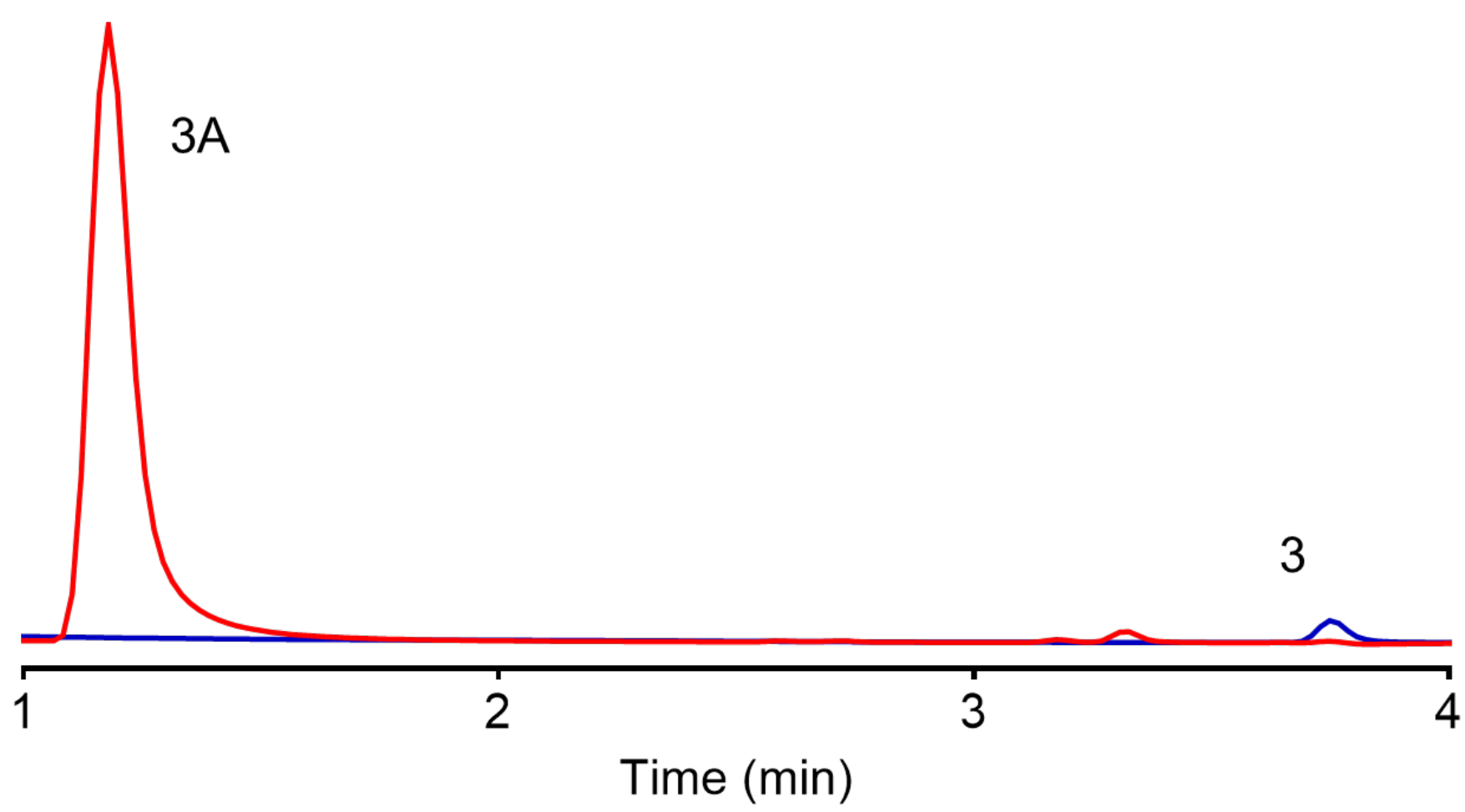

Figure S. 8: UPLC trace for the amination of Compound **3** by RTA-223. Red:biotransformation, Blue:standard of **3**.

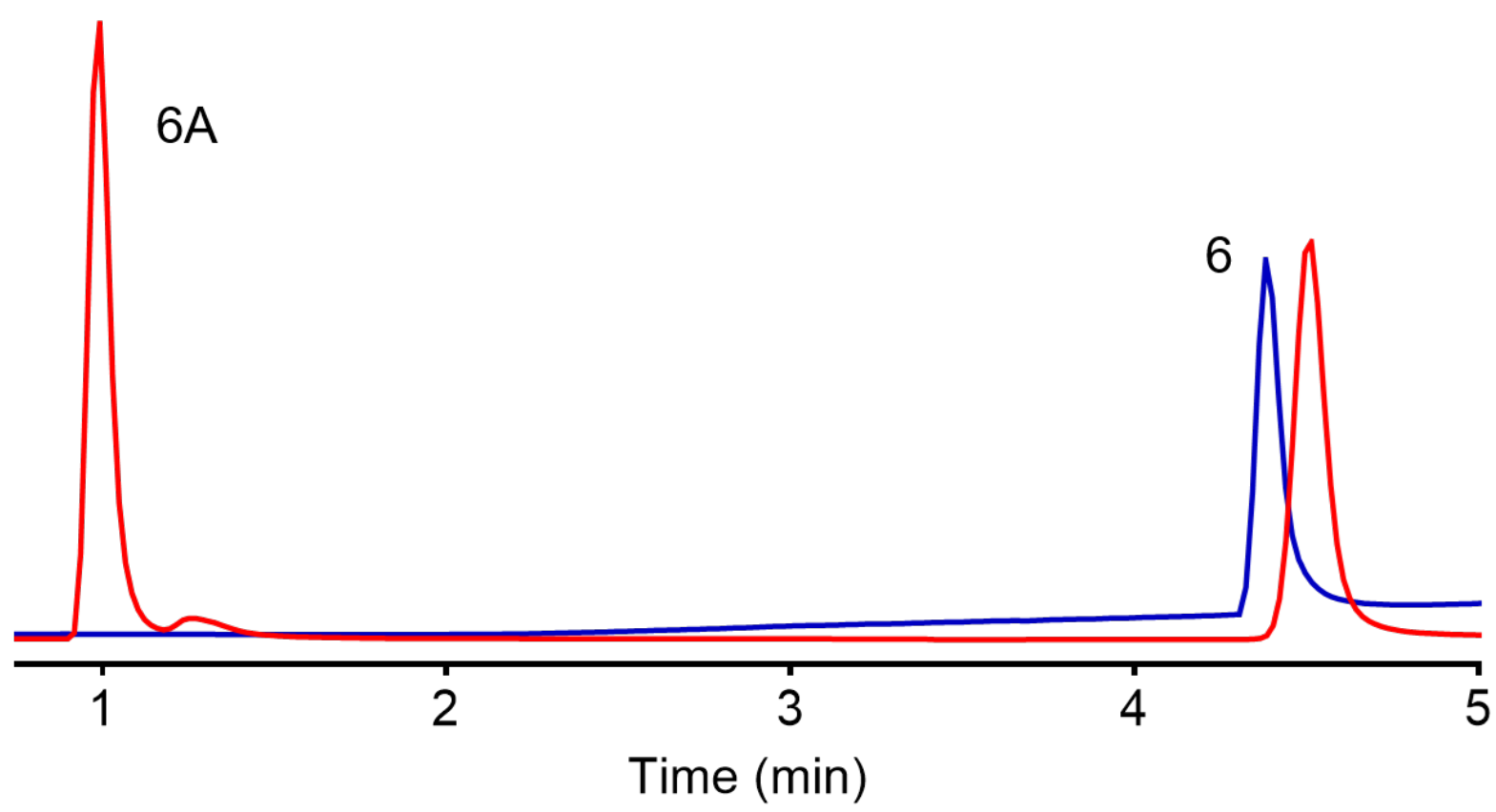

Figure S. 9: UPLC trace for the amination of Compound **6** by RTA-223. Red: biotransformation, Blue: Standard of **6**.

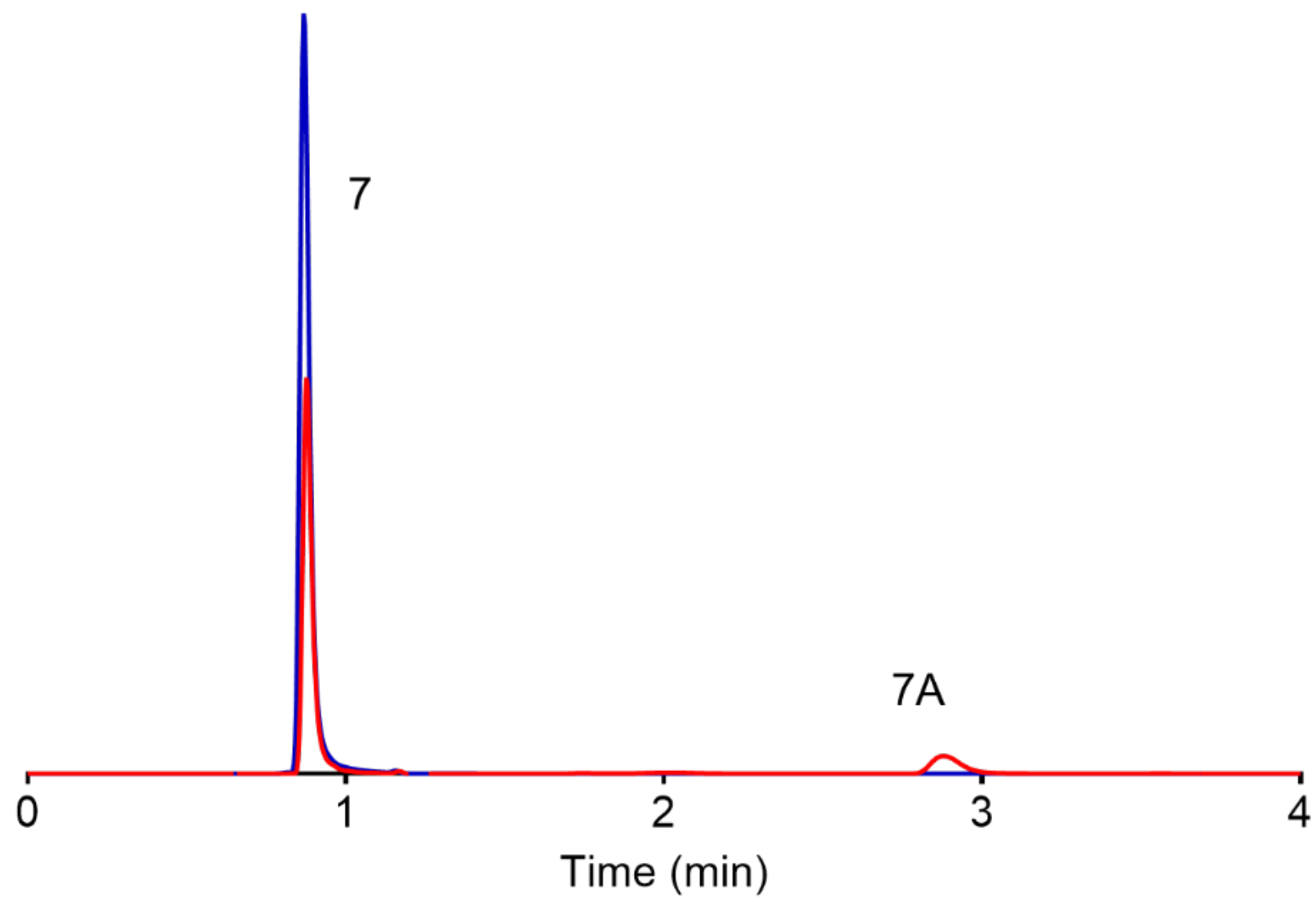

Figure S. 10: UPLC trace for the amination of Compound **7** by RTA-223. Red: biotransformation, Blue: standard of **7**.

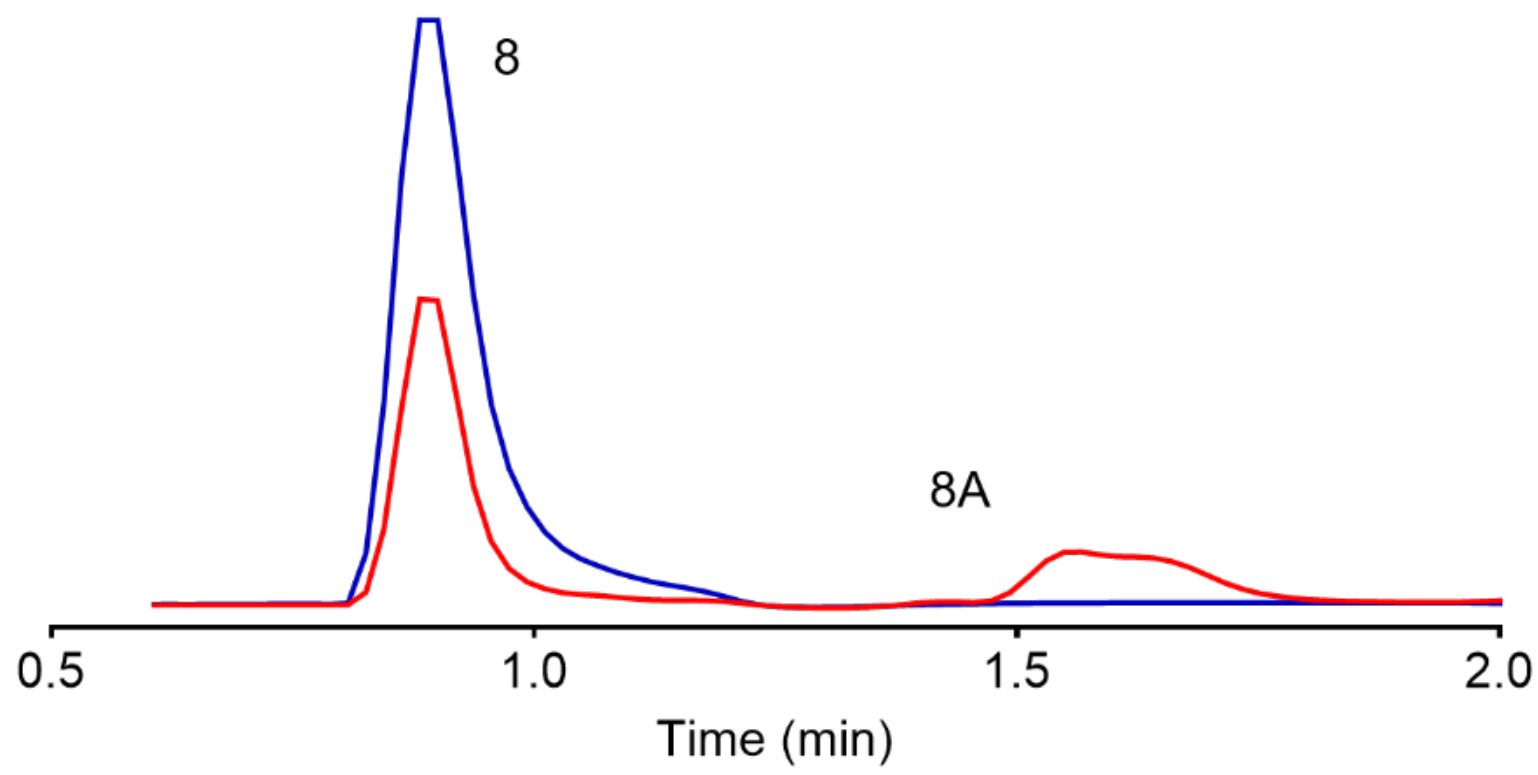

Figure S. 11: UPLC trace for the amination of compound **8**. Red: biotransformation, Blue: standard of **8**.

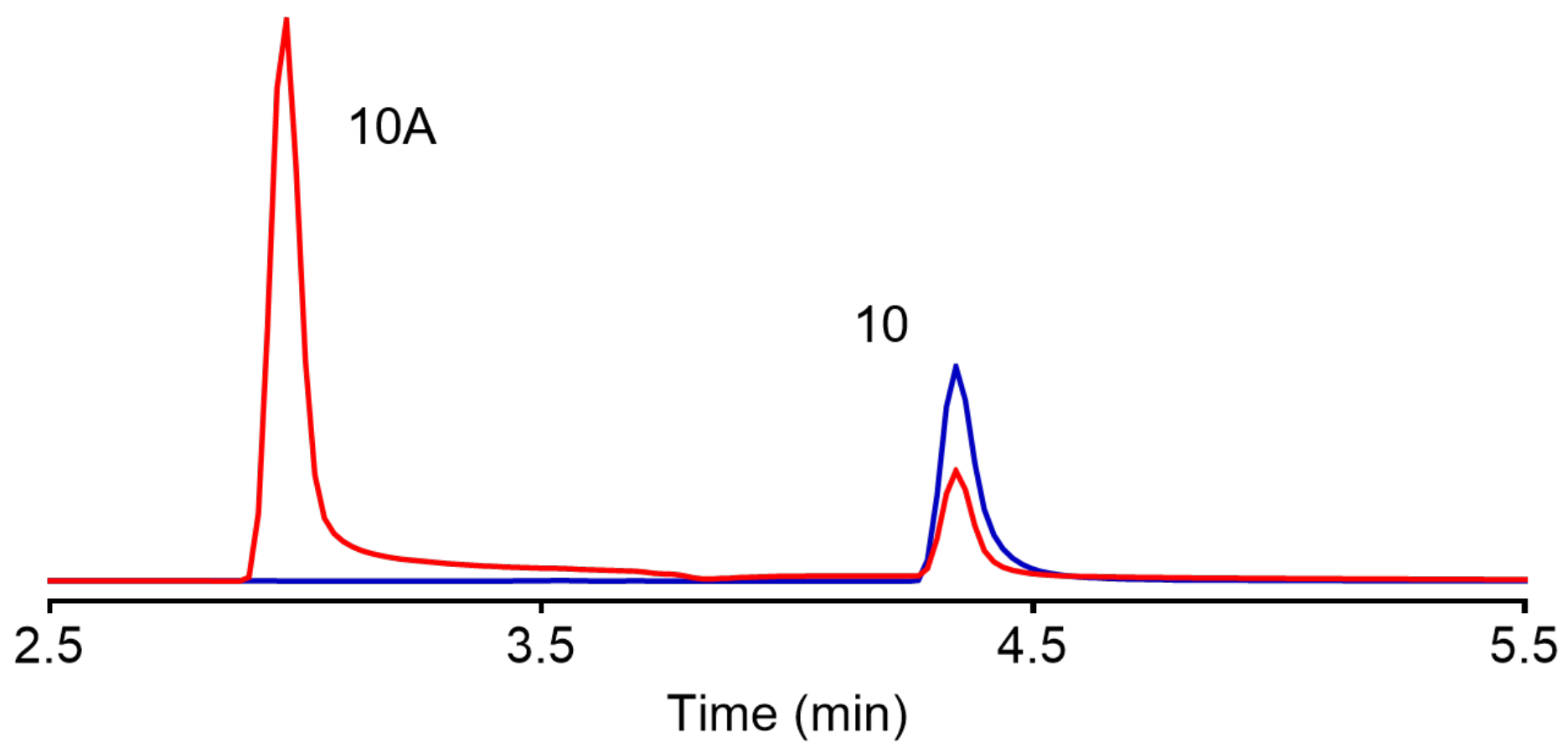

Figure S. 12: UPLC trace for the amination of compound **10**. Red: biotransformation, Blue: standard of **10**.

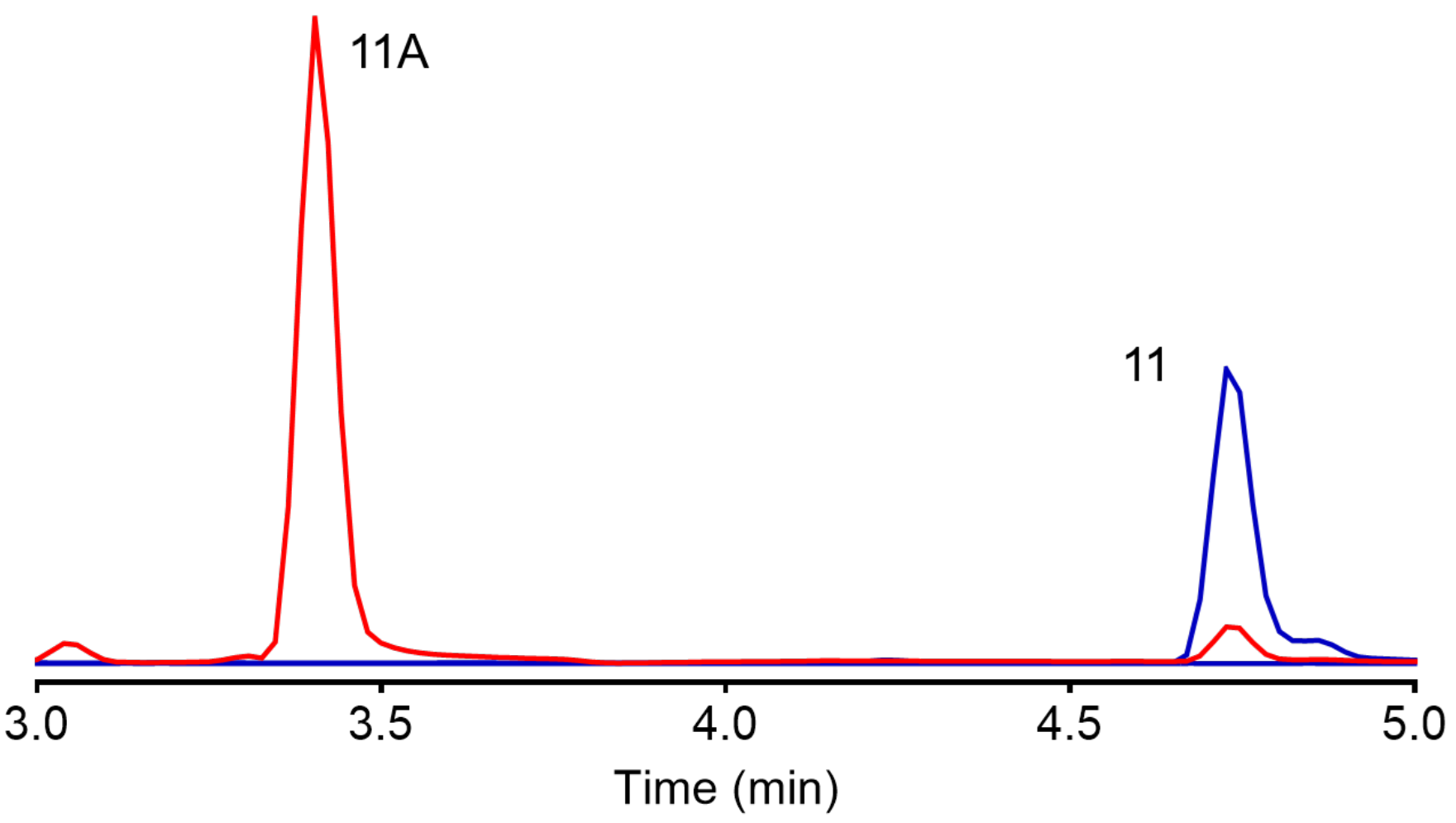

Figure S. 13: UPLC trace for the amination of compound **11**. Red: biotransformation, Blue: standard of **11.**

5.2 Data to determine enantiomeric ratio

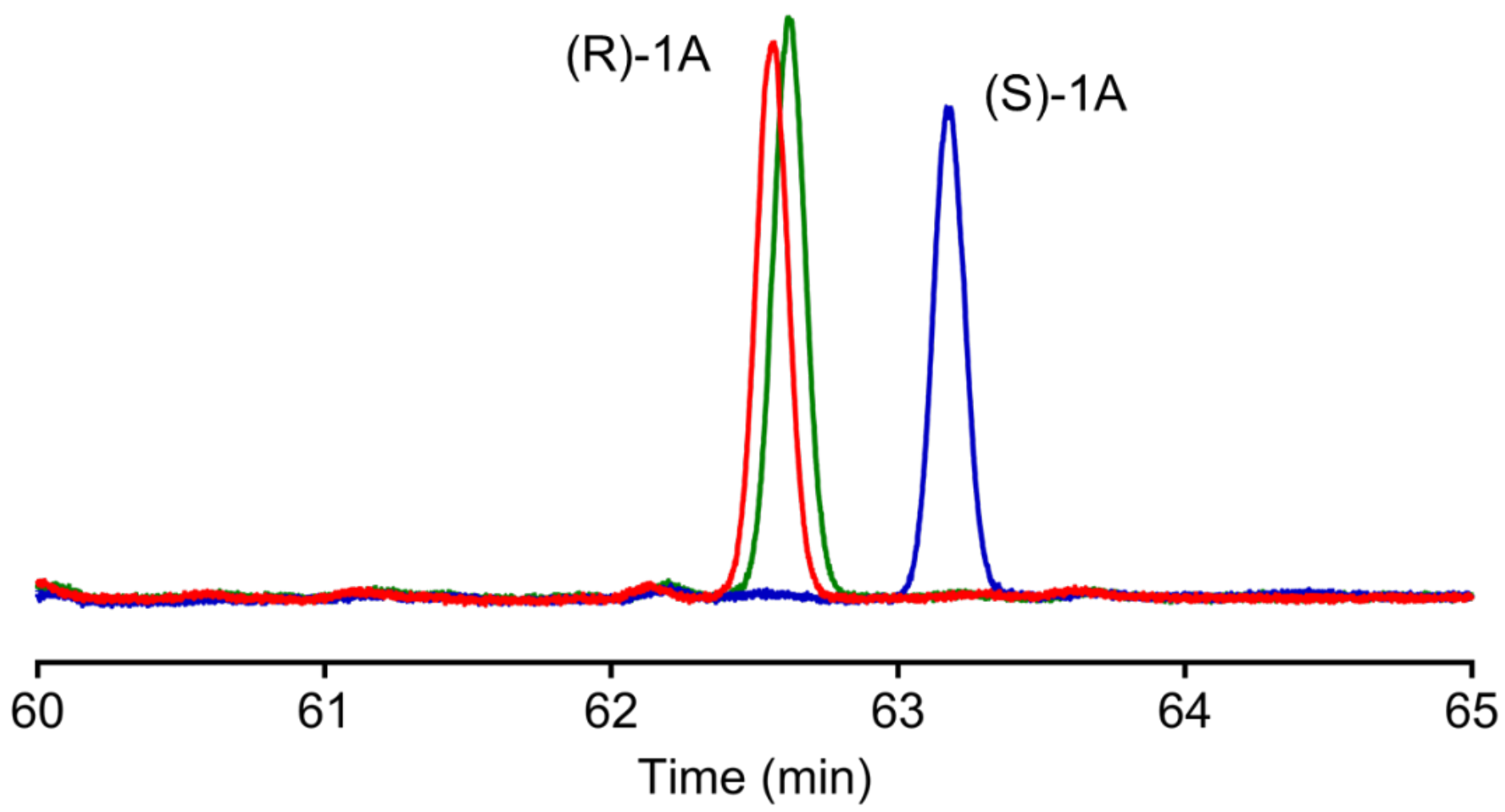

Figure S. 14: Chiral GC trace of reductive amination of Compound **1** by RTA-223. Red: biotransformation, Blue: (*S*)-α-methylbenzylamine standard, Green: (*R*)-α-methylbenzylamine standard

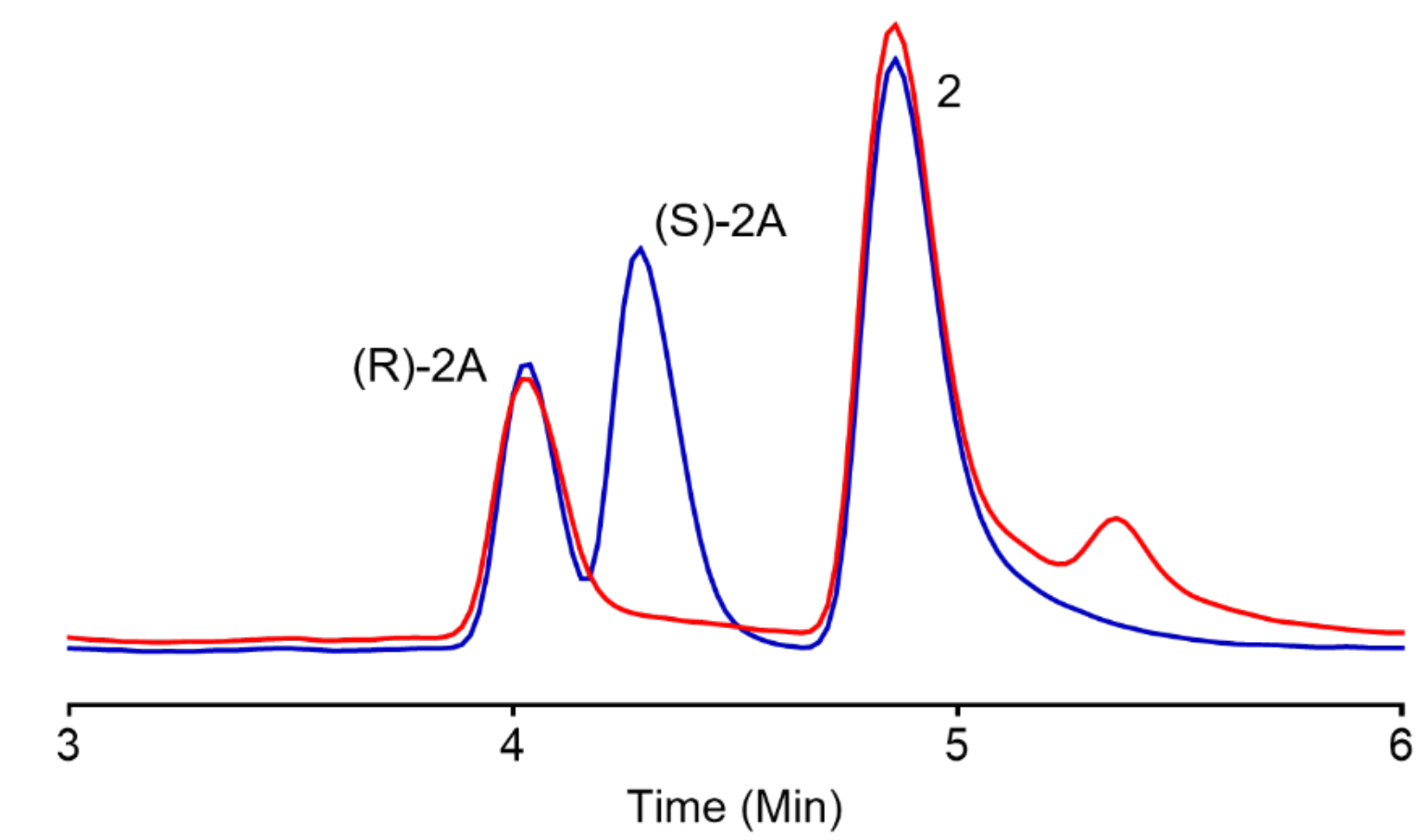

Figure S. 15: Chiral HPLC for the reductive amination of Compound **2** by RTA-223. Red: biotransformation, Blue: non-selective chemical reductive amination of Compound **2**.

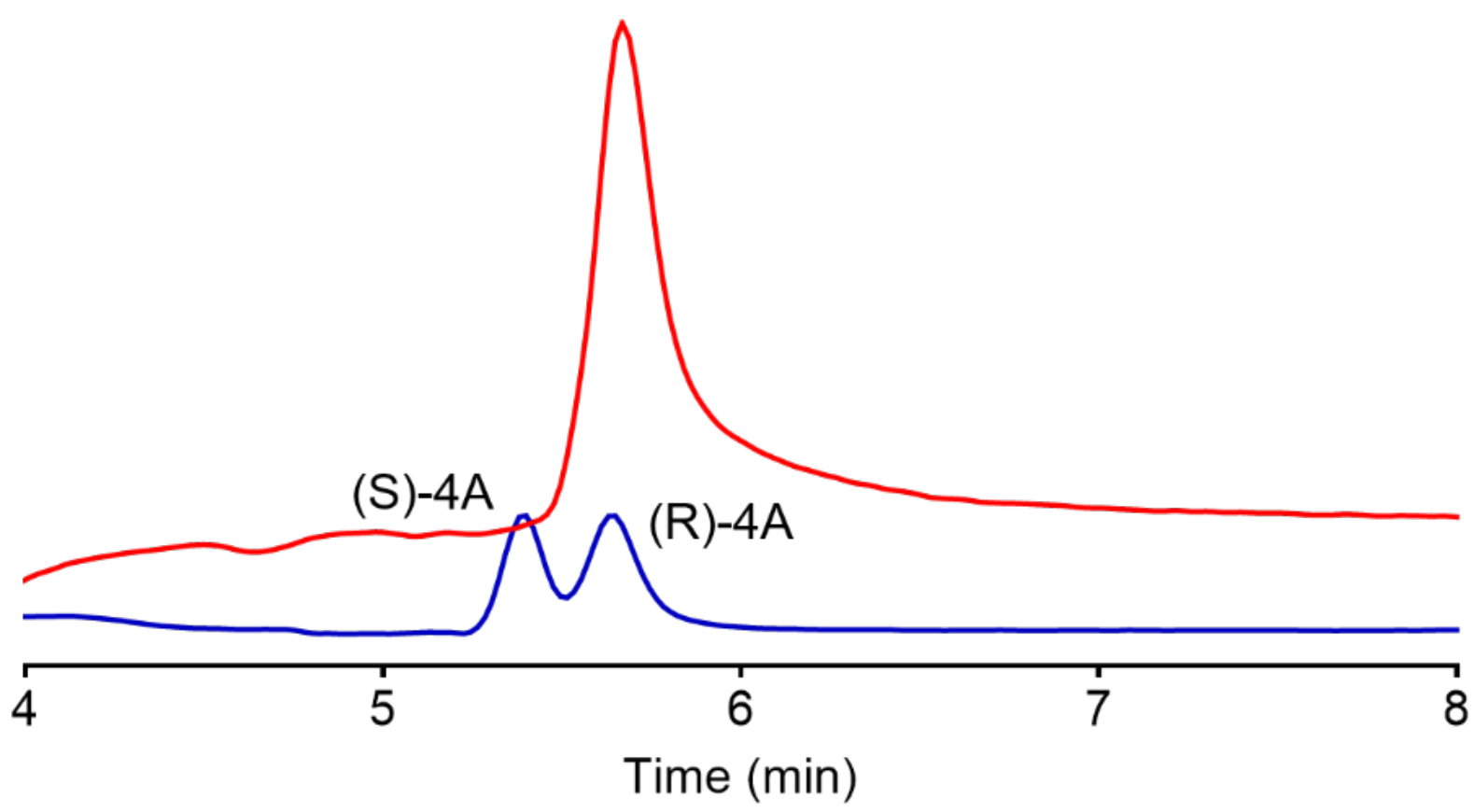

Figure S. 16: Chiral HPLC for the reductive amination of Compound **4** by RTA-223. Red: biotransformation, Blue: non-selective chemical reductive amination of Compound **4**.

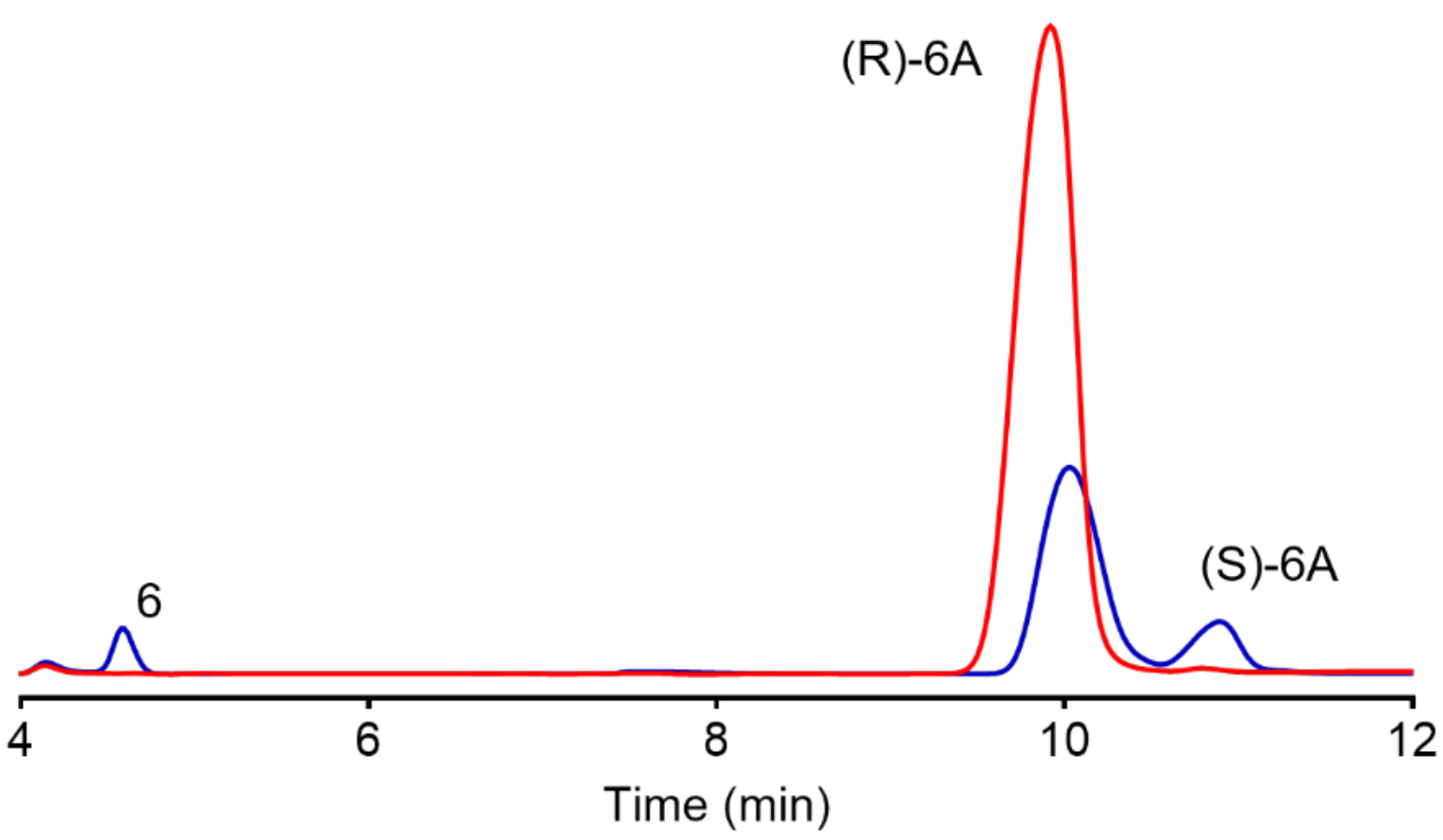

Figure S. 17: Chiral HPLC for the reductive amination of Compound **6** by RTA-223. Red: biotransformation, Blue: non-selective chemical reductive amination of Compound **6**.

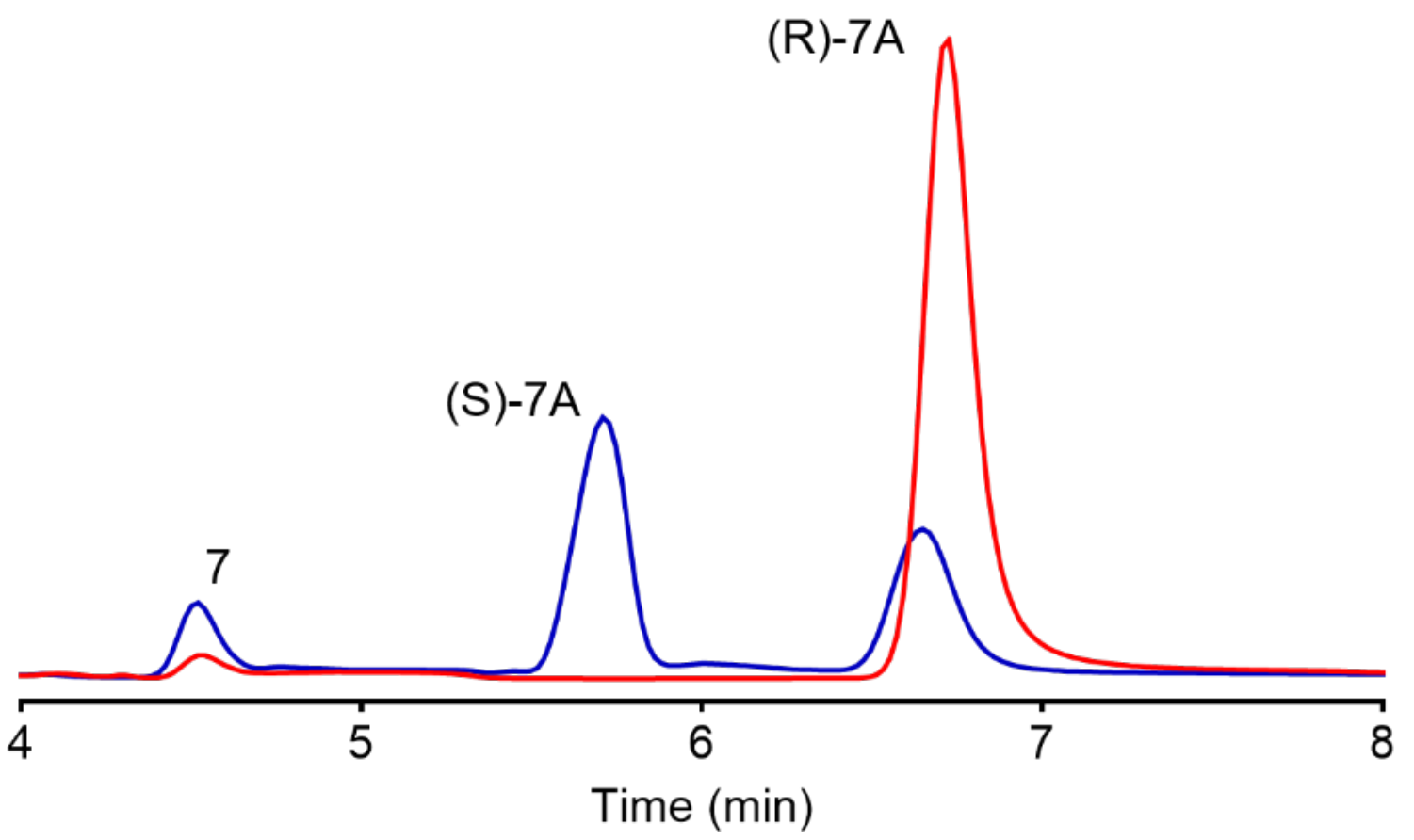

Figure S. 18: Chiral HPLC for the reductive amination of Compound **7** by RTA-223. Red: biotransformation, Blue: non-selective chemical reductive amination of Compound **7**.

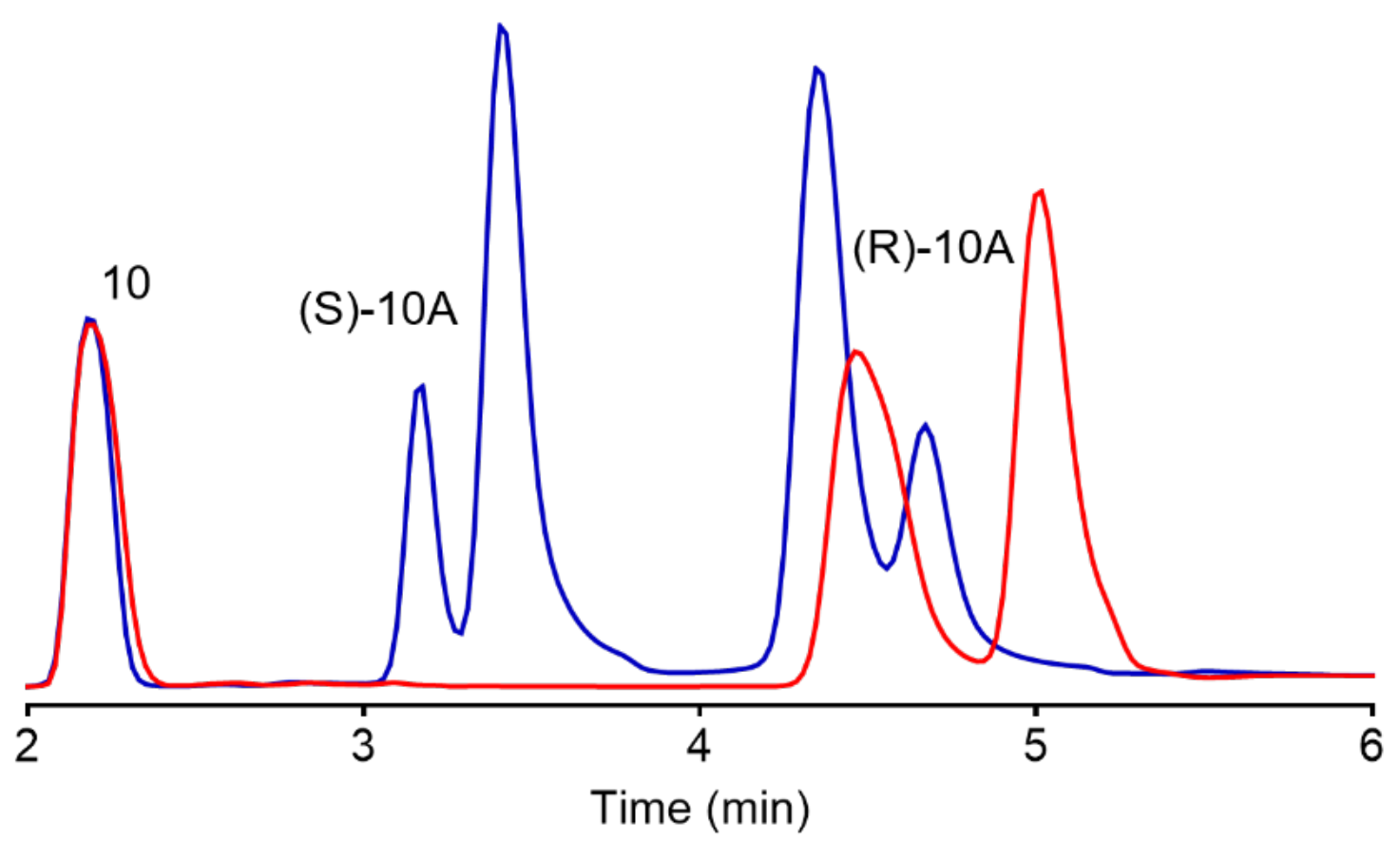

Figure S. 19: Chiral HPLC for the reductive amination of Compound **10** by RTA-223. Red: biotransformation, Blue: non-selective chemical reductive amination of Compound **10**. Note that the individual diastereomers could not be unambiguously assigned.

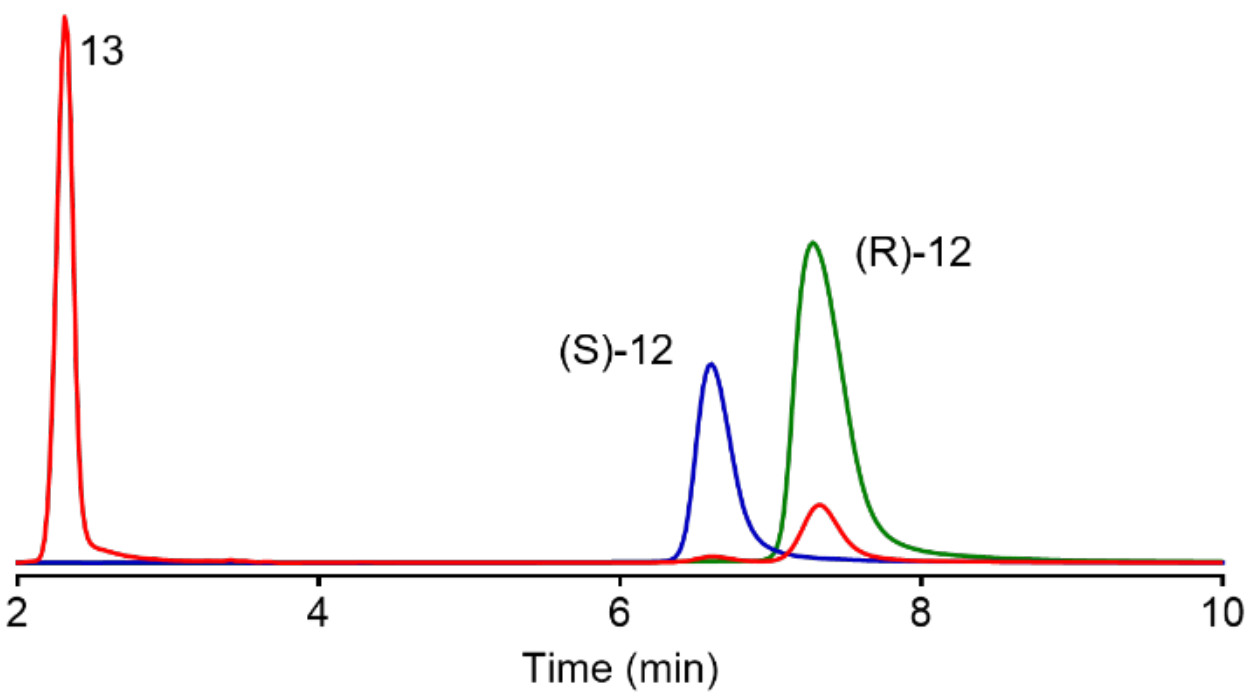

Figure S. 20: Chiral HPLC for the conversion of **13** to **(*R*)-12** by RTA-223 (H53L/V60G/F113A/V148A)**.** Red: biotransformation, Blue: **(*S*)-12** standard, Green: **(*R*)-12** standard

6. Sites targeted during evolution

Table S.1: Sites targeted during each round of evolution, along with the parent gene used in each round

Round of evolution	Parent gene	Sites targeted
Round 1	F113A	Q49 Y58 H74 R77 V114 E115 R126 F141 Q143 I146 V148 K179 W183 N217 V237 T238 T274
Round 2	F113A/V148A	F51 H54 V60 W64 F69 R70 D73 I109 A113 V114 W147 Y200 F216, P229 T238 K240 F270 Y305

7. Fluorescence assay to determine transaminase rate

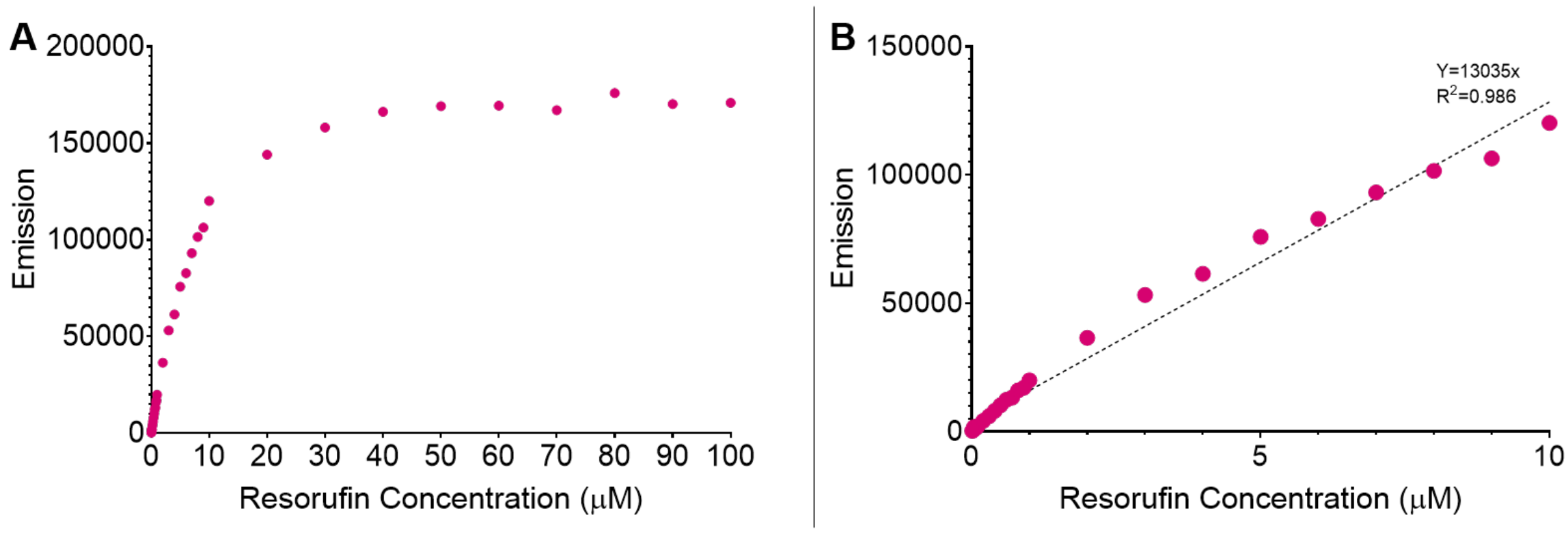

Figure S. 21: Resorufin calibration curves. **A:** Calibration curve showing the maximum detection. **B:** Curve fitting up to 10 μM resorufin.

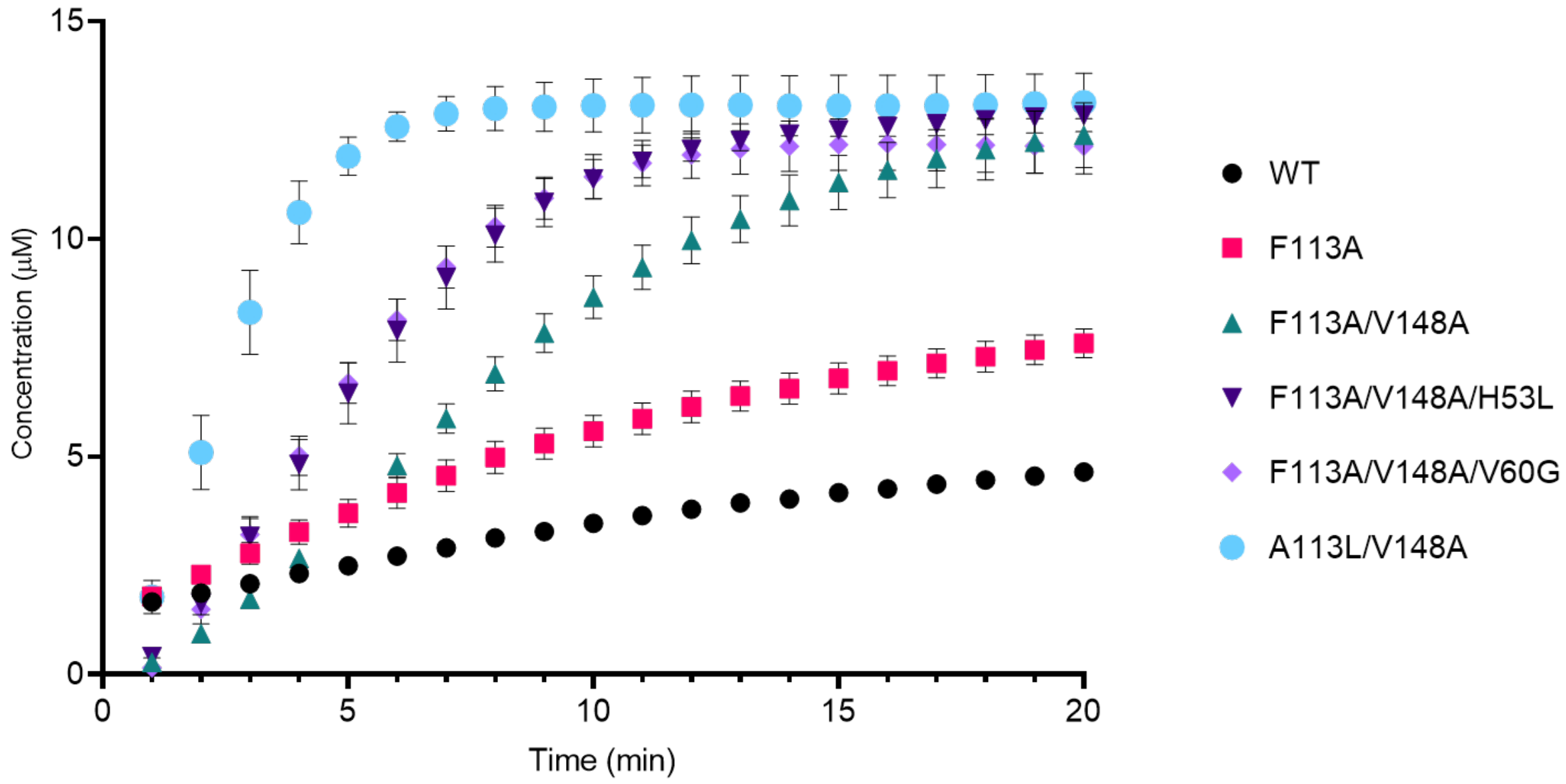

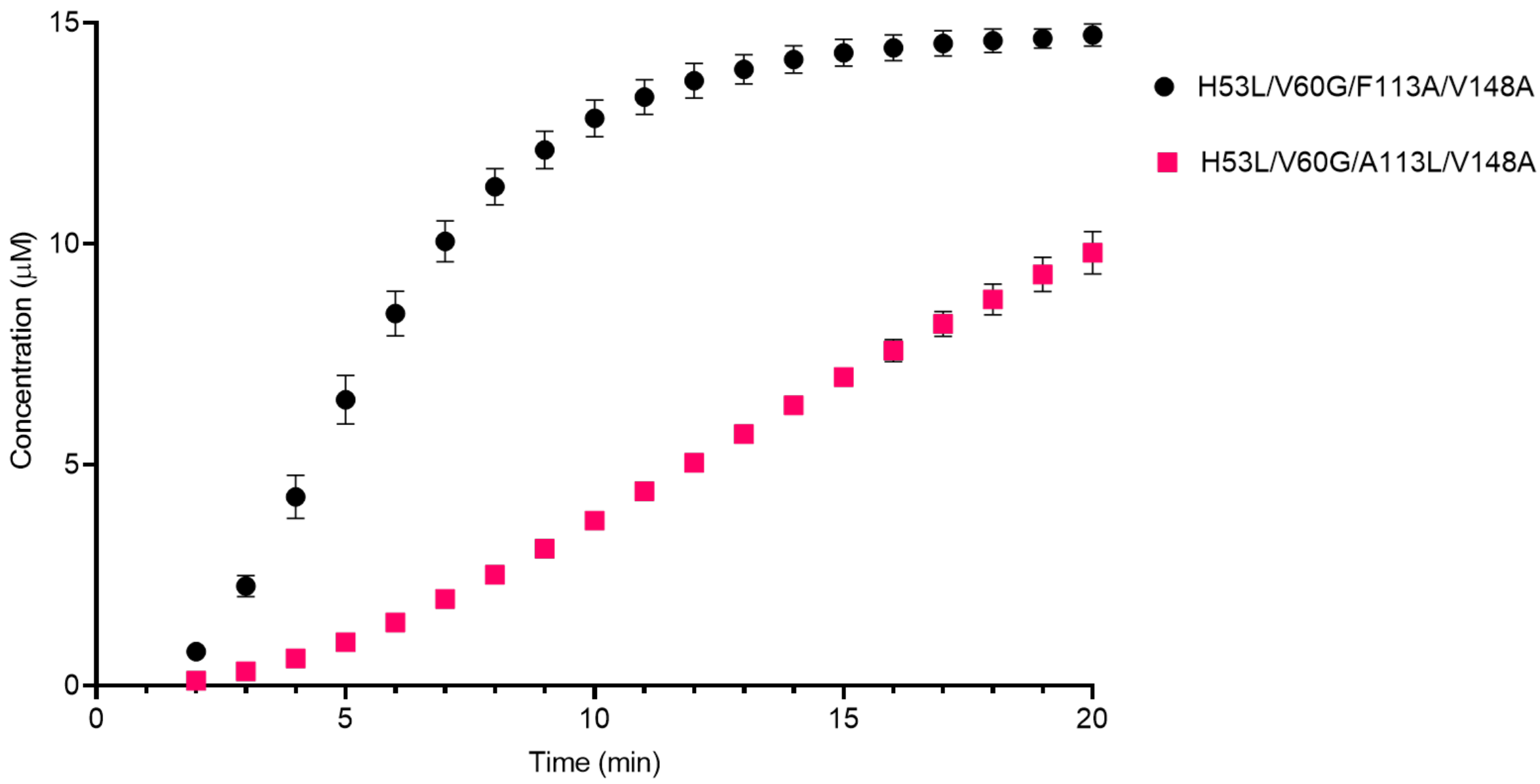

Figure S. 22: Kinetic curves from the coupled fluorescence assay to determine the rate of pro-sitagliptin amination using: Top 2.5 mg/mL, Bottom: 1.0 mg/mL of RTA-223 variants.

Table S. 2: Data points fitted from Figures S21 and S22 to yield rates.

RTA-223 variant	Points fitted (min)	Rate (μM/min/mg)
WT	1-10	1.62
F113A	1-10	3.42
F113A/V148A	1-10	7.78
H53L/F113A/V148A	1-10	7.69
V60G/F113A/V148A	1-10	10.54
A113L/V148A	1-5	20.60
H53L/V60G/A113L/V148A	1-10	9.19
H53L/V60G/F113A/V148A	1-10	31.60

8. Primers

Table S. 3: Primers for NNK site saturation libraries.

Primer	SEQUENCE
ROUND 1	
49ss_F	ATTCCGCTGCTGGATNNKGGTTTCTTACATAGTGACCTTAC
49_R	ATCCAGCAGCGGAAT
58ss_F	CATAGTGACCTTACCNNKGATGTACCAGCTGTTTGG
58_R	GGTAAGGTCACTATGTAAGAAAC
74ss_F	TTCCGCTTGGATGATNNKCTTAGCCGCCTTGAGA
74_R	ATCATCCAAGCGGAAAAAAC
77ss_F	GATGATCACCTTAGCNNKCTTGAGAAAAGCTGTTCGAA
77_R	GCTAAGGTGATCATCCAAG
114ss_F	ATTCGGGACGTGTTTNNKGAGTTAATTGTTACTAGAGGTCTG
114_R	AAACACGTCCCGAATAC
115ss_F	CGGGACGTGTTTGTTNNKTTAATTGTTACTAGAGGTCTGAAGG
115_R	AACAAACACGTCCCG
141ss_F	AACACCCTGTACATGNNKATCCAGCCCTATATCTGG
141_R	CATGTACAGGGTGTTGAC
126ss_F	GGTCTGAAGGGCGTCNNKGAAGCGAAAAATCCTGAGG
126_R	GACGCCCTTCAGACC
143ss_F	CTGTACATGTTTATCNNKCCCTATATCTGGGTAATGGA
143_R	GATAAACATGTACAGGGTGTTG

146ss_F	TTTATCCAGCCCTATNNKTGGGTAATGGAACCTGAA
146_R	ATAGGGCTGGATAAACATGT
148ss_F	CAGCCCTATATCTGGNNKATGGAACCTGAAGTGCA
148_R	CCAGATATAGGGCTGGAT
179ss_F	ATGGACCCGACGGTTNNKAACCTGCAATGGGGC
179_R	AACCGTCGGGTCC
183ss_F	GTTAAAAACCTGCAANNKGGCGATCTCGTGC
183_R	TTGCAGGTTTTTAACCGTC
217ss_F	GAAGGATCCGGTTTCNNKATTGTGTTAGTCAAAGATGGG
217_R	GAAACCGGATCCTTCTG
237ss_F	GGTGTACTGGAAGGGNNKACACGAAAAAGTGTGTTCG
237_R	CCCTTCCAGTACACCT
238ss_F	GTACTGGAAGGGGTGNNKCGAAAAAGTGTGTTCGATGT
238_R	CACCCCTTCCAGTACA
274ss_F	ATTTTTATGTGCACANNKGCGGGCGGCATTA
274_R	TGTGCACATAAAAATCTCATCAG
Round 2	
109_R	ACCACTTTTCGTCACCAT
109ss_F	GTGACGAAAAGTGGTNNKCGGGACGTGGCT
114_R	AGCCACGTCCCG
114ss_F	ATTCGGGACGTGGCTNNKGAGTTAATTGTTACTAGAGGTCTG
145_R	GGGCTGGATAAACATGTAC
145ss_F	ATGTTTATCCAGCCCNNKATCTGGGCTATGGAACC
147_R	GATATAGGGCTGGATAAACATG
147ss_F	ATCCAGCCCTATATCNNKGCTATGGAACCTGAAGTG
200_F	GATCGAGGGGCTGCGNNKCCGTTTCTCACCGATG
200_R	CGCAGCCCCTCG
216_R	ACCGGATCCTTCTGTTATATT
216ss_F	ACAGAAGGATCCGGTNNKAACATTGTGTTAGTCAAAGATGG
229_R	GGTGTAGATTACCCCATCT
229ss_F	GGGGTAATCTACACCNNKGATAGAGGTGTACTGGAAGG
238_R	CACCCCTTCCAGTACA
238ss_F	GTACTGGAAGGGGTGNNKCGAAAAAGTGTGTTCGATGT
240_R	TCGTGTCACCCCTTC
240ss_F	GAAGGGGTGACACGANNKAGTGTGTTCGATGTTGC

270_R	AATCTCATCAGCCTGATAGG
270ss_F	CAGGCTGATGAGATTNNKATGTGCACAACTGCG
305_R	GTCATCCCAGATTTTCTGG
305ss_F	AAAATCTGGGATGACNNKTGGGCCTTACATTATGATCC
51_R	ACCCTGATCCAGCAG
51ss_F	CTGCTGGATCAGGGTNNKTTACATAGTGACCTTACCTACG
64_R	AACAGCTGGTACATCGTA
64ss_F	GATGTACCAGCTGTTNNKGATGGGCGTTTTTTCCG
69_R	AAAACGCCCATCCCA
69ss_F	TGGGATGGGCGTTTTNNKCGCTTGGATGATCACC
70_R	GAAAAAACGCCCATCGG
70ss_F	GATGGGCGTTTTTTCNNKTTGGATGATCACCTTAGCC
73_R	ATCCAAGCGGAAAAAACG
73ss_F	TTTTTCCGCTTGGATNNKCACCTTAGCCGCCT
